# A taxonomic revision and molecular phylogeny of the eastern Palearctic species of the genera *Schizomyia* Kieffer and *Asteralobia* Kovalev (Diptera, Cecidomyiidae, Asphondyliini), with descriptions of five new species of *Schizomyia* from Japan

**DOI:** 10.3897/zookeys.808.29679

**Published:** 2018-12-18

**Authors:** Ayman Khamis Elsayed, Junichi Yukawa, Makoto Tokuda

**Affiliations:** 1 The United Graduate School of Agricultural Sciences, Kagoshima University, Kagoshima 890–0065, Japan Kagoshima University Kagoshima Japan; 2 Laboratory of Systems Ecology, Faculty of Agriculture, Saga University, Saga 840–8502, Japan Saga University Saga Japan; 3 Department of Applied Entomology, Faculty of Agriculture, Alexandria University, Alexandria, Egypt Alexandria University Alexandria Egypt; 4 Entomological Laboratory, Faculty of Agriculture, Kyushu University, Fukuoka 819–0395, Japan Kyushu University Fukuoka Japan

**Keywords:** Cecidomyiinae, gall midges, Schizomyiina, taxonomic key

## Abstract

The genus *Asteralobia* (Diptera, Cecidomyiidae, Asphondyliini, Schizomyiina) was erected by Kovalev (1964) based on the presence of constrictions on the cylindrical male flagellomeres. In the present study, we examine the morphological features of *Asteralobia* and *Schizomyia* and found that the male flagellomeres are constricted also in *Schizomyiagaliorum*, the type species of *Schizomyia*. Because no further characters clearly separating *Asteralobia* from *Schizomyia* were observed, we synonymize *Asteralobia* under *Schizomyia*. Molecular phylogenetic analysis strongly supports our taxonomic treatment. We describe five new species of *Schizomyia* from Japan, *S.achyranthesae* Elsayed & Tokuda, **sp. n.**, *S.diplocyclosae* Elsayed & Tokuda, **sp. n.**, *S.castanopsisae* Elsayed & Tokuda, **sp. n.**, *S.usubai* Elsayed & Tokuda, **sp. n.**, and *S.paederiae* Elsayed & Tokuda, **sp. n.**, and redescribe three species, *S.galiorum* Kieffer, *S.patriniae* Shinji, and *S.asteris* Kovalev. A taxonomic key to the Japanese *Schizomyia* species is provided.

## Introduction

*Schizomyia* Kieffer is a cosmopolitan genus of the subtribe Schizomyiina (Diptera, Cecidomyiidae, Cecidomyiinae, Asphondyliini) with 53 species associated with diverse plant families (Gagné and Jaschhof 2017, Elsayed et al. in press). The genus includes species with needle-like ovipositors, four-segmented palpi, and larval terminal segments with four or fewer pairs of terminal papillae (Gagné and Menjivar 2008). Most of these species induce bud galls, but some induce leaf galls (Gagné 1989). Some *Schizomyia* species are agricultural pests, e.g. *S.loroco* Gagné, which induces flower galls on loroco, *Fernaldiapandurata* (A. DC.) Woodson (Apocynaceae), in El Salvador (Gagné and Menjivar 2008). A few species have been used as potential biological control agents, e.g., *S.macrofila* (Felt), which induces flower galls on *Amsinckia* spp. weeds in California (Pantone and Brown 1985).

Kovalev (1964), in his revision of the Russian Far East gall midges of the tribe Asphondyliini, erected the genus *Asteralobia* based on the presence of shallow or deep constrictions on the cylindrical male flagellomeres. Although *Asteralobia* has been treated as an independent genus of Schizomyiina and presently contains 12 species (Gagné and Jaschhof 2017), some studies have indicated that *Asteralobia* could be subsumed under *Schizomyia* because of the lack of known synapomorphic differences between them (e.g. Tokuda et al. 2003; Gagné and Jaschhof 2017). In the present study, we re-examined morphological features of *Asteralobia* and *Schizomyia* and analyzed molecular phylogenetic relationships between them, which have led us to the conclusion that *Asteralobia* should be synonymized under *Schizomyia*. In addition, we describe five new species of *Schizomyia* from Japan and provide a taxonomic key to the *Schizomyia* species from Japan.

## Materials and methods

### Collecting and rearing methods

Galls induced by six gall midge species were collected from various localities in Japan (Table 1). Some galls were dissected to obtain larval specimens, while the remaining galls were kept in plastic bags until the departure of mature larvae from the galls. Thereafter, larvae were transferred to plastic cups (120 mm in diameter, 110 mm in depth) containing a mixture of wet peat moss and sand (1:1), covered with fine mesh, and fixed with a rubber band. Cups containing larvae obtained from fruit galls on *Achyranthesbidentata* Blume (Amaranthaceae) and *Trachelospermumasiaticum* (Sieb. et Zucc.) Nakai (Apocynaceae), and flower bud galls on *Paederiafoetida* L. (Rubiaceae) and *Patriniavillosa* (Thunb.) (Valerianaceae) were transferred to the field on the Saga University campus, Saga Prefecture (about 5.5 m a.s.l.), and half-buried in the soil to allow the mature larvae to overwinter under natural conditions. These cups were brought back to the laboratory in April to rear adults and were kept at room temperature. In cases of larvae obtained from flower bud galls on *Diplocyclospalmatus* (L.) Jeffrey (Cucurbitaceae) and *Castanopsissieboldii* (Makino) Hatus. (Fagaceae), the cups containing larvae were maintained at room temperature in the laboratory until adult emergence. The pupal exuviae protruding from the surface of the soil in the plastic cups were collected at the same time carefully. Reared specimens were preserved in 75% ethanol for morphological study and 99.5% ethanol for the molecular phylogenetic study.

**Table 1.** Collection data of the newly described and redescribed Japanese *Schizomyia* species.

Gall midge	Host Plant and galls	Collection site	Collecting date	Collector
*Schizomyiaachyranthesae* sp. n.	Fruit bud galls on *Achyranthesbidentata* Blume (Amaranthaceae)	Tokushima City, Tokushima Prefecture	6 October 2001	M. Yukinari
		Kyushu University, Ito Campus, Fukuoka Prefecture	30 October 2012	J. Yukawa et al.
		Mount Hinokuma, Saga Prefecture	16 October 2014	A.K. Elsayed & M. Tokuda
		Mount Tara, Saga Prefecture	22 October 2014	A.K. Elsayed & M. Tokuda
		Mount Tenzan, Saga Prefecture	29 October 2001	A.K. Elsayed & M. Tokuda
		Mount Tara, Saga Prefecture	9 October 2015	M. Tokuda
		Takeo City, Saga Prefecture	10 October 2015	A. Kita
*Schizomyiadiplocyclosae* sp. n.	Flower bud galls on *Diplocyclospalmatus* (L.) Jeffrey (Cucurbitaceae)	Kinjo town, Naha City, Okinawa Prefecture	13 January 1977	S. Yamauchi
		Shuri, Naha City, Okinawa Prefecture	January 1977	S. Yamauchi
		Gogayama, Nakijin village, Okinawa Prefecture	4 March 2002	M. Tokuda
		Hantagawa, Naha City, Okinawa Prefecture	10 February 2016	T. Ganah-Kikumura
*Schizomyiapaederiae* sp. n.	Flower bud galls on *Paederiafoetida* L. (Rubiaceae)	Nishino-omote, Nishino-omote City, Nokubi, Kagoshima Prefecture	24 September 2014	K. Ogata
		Ogorori City, Misawa, Aomori Prefecture	August 2016	K. Matsunaga
*Schizomyiapatriniae* (Shinji 1938)	Flower bud galls on *Patriniavillosa* (Thunb.) (Valerianaceae)	Kyuragi, Karatsu City, Saga Prefecture	12 October 2015	M. Tokuda
*Schizomyiacastanopsisae* sp. n.	Flower bud galls on *Castanopsissieboldii* (Fagaceae)	Hachijojima Island	6 December 2014	T. Kikuchi
		Shikinejima Island	10 December 2014	M. Tokuda
		Niijima Island	11 December 2014	M. Tokuda
		The Izu Islands	9 December 2016	M. Tokuda
*Schizomyiausubai* sp. n.	Fruit galls on *Trachelospermumasiaticum* (Sieb. et Zucc.) Nakai (Apocynaceae)	Mount Takakuma, Kagoshima Pref.	November 1969	J. Yukawa
		Imuta Lake-side, Kedouin Town, Kagoshima Prefecture	2 November 1978	S. Sako
		Torinosu, Tanabe City, Wakayama Prefecture	2 November 2016	I. Matoba
		Mount Onigasawa, Nishino-omote, Nishino-omote City, Kagoshima Prefecture	4 November 2016	K. Ogata

### Taxonomy

Gall midge specimens were mounted on slides in Canada balsam using the technique outlined in Gagné (1994), except for the clearing step following Elsayed et al. (2018). The slide-mounted specimens were examined under a bright-field and phase-contrast microscope (H550L, Nikon, Tokyo) and illustrations were made with the aid of a drawing tube. A semi-motorized fluorescence microscope (BX53, Olympus, Tokyo) was used to photograph some of the characters from mounted specimens with the aid of a microscope-attached camera (DP22, Olympus, Tokyo).

Morphological terminology basically follows McAlpine et al. (1981) for adults, but the thoracic sclerite “mesanepisternum” follows Gagné (1968) and the wing venation follows Yukawa (1971). Larval and pupal terminology follows Gagné (1994). All the types of the newly described species are deposited in the collection of the Entomological Laboratory, Faculty of Agriculture, Kyushu University, Japan (KUEC).

Adult and immature specimens of *Asteralobiahumuli* Shinji, *A.patriniae* Shinji, *A.sasakii* Monzen and *A.soyogo* Kikuti, and larvae of *A.doellingeriae* Kovalev were examined in KUEC. Adults of *A.asteris* Kovalev, *A.calathidiphaga* Kovalev, *A.doellingeriae*, and *A.solidaginis* Kovalev, as well as four females and two males of *S.galiorum* were examined in the B. M. Mamaev Collection in the Museum of Nature and Human Activities, Hyogo, Japan. A female and a pupa of *Schizomyiagaliorum* Kieffer were examined in the National Museum of Natural History, Smithsonian Institution, Washington, DC, USA (USNM).

### Molecular phylogenetic study

Total DNA was extracted from larval or adult specimens and fragments of the mitochondrial genes, cytochrome oxidase subunit I (COI) and 12S small ribosomal subunit, were sequenced and aligned following Elsayed et al. (2017). The following sets of primers were used for the cytochrome oxidase subunit I (COI) gene: J–1718 (5'–GGA GGA TTT GGA AAT TGA TTA GTT CC–3') (Simon et al. 1994) and COIA (5'–CCC GGT AAA ATT AAA ATA TAA ACT TC–3’) (Funk et al. 1995); COIS (5–GGA TCA CCT GAT ATA GCA TTC CCA TAT TGG–3’) and COIA (5'–CCC GGT AAA ATT AAA ATA TAA ACT TC–3’) (Funk et al., 1995); or LCO1490 (5'–GGT CAA CAA ATC ATA AAG ATA TTG G–3') and HCO2198 (5'–TAA ACT TCA GGG TGA CCA AA AAT CA–3') (Folmer et al. 1994). The following primer set was used for the 12S small ribosomal subunit gene: SR–J–14199 (5'–TAC TAT GTT ACG ACT TAT–3’) and SR–N–14594 (5'–AAA CTA GGA TTA GAT ACC C–3’) (Kambhampati and Smith 1995). The obtained sequences were deposited in the DNA Data Bank of Japan (DDBJ), the European Molecular Biology Laboratory (EMBL), and GenBank under the accession numbers given in Table 2.

**Table 2.** Gall midge species used for genetic analysis.

Gall midge	Host Plant	Collection site	Collector	*COI* accession no.	*12S* accession no.
*Schizomyiaachyranthesae* sp. n.	*Achyranthesbidentata* Blume (Amaranthaceae)	Mount Tara, Saga Pref., Japan	M. Tokuda	LC426387–LC426389	LC426410-LC426412
*S.* (=*Asteralobia*) *asteris* (Kovalev)	*Astertataricus* L. fil. (Asteraceae)	Smolyaninovo, Primorsky Territory, Russia	M. Tokuda et al.	LC426390	LC426413
*S.buboniae* (Frauenfeld)	*Deverratortuosa* (Desf.) DC. (Apiaceae)	Borg Al-Arab District, Alexandria, Egypt	A.K. Elsayed	LC426391-LC426393	LC426414-LC426416
*S.* (=*Asteralobia*) *doellingeriae* (Kovalev)	*Asterscaber* Thunb. (Asteraceae)	Shkotovo, Primorsky Territory, Russia	M. Tokuda	LC422074 ^¶^	LC422101 ^¶^
*S.diplocyclosae* sp. n.	*Diplocyclospalmatus* (L.) Jeffrey (Cucurbitaceae)	Hantagawa, Naha City, Okinawa Pref., Japan	T. Ganaha-Kikumura	LC426394-LC426396	LC426417-LC426419
*S.* (=*Asteralobia*) *kovalevi* (Skuhravá)	*Patriniascabiosifolia* Fisch. (Valerianaceae)	Dukhovskoye, Primorsky Territory, Russia	M. Tokuda et al.	LC422068 ^¶^	LC422095 ^¶^
*S.* (=*Asteralobia*) *patriniae* (Shinji)	*Patriniavillosa* (Thunb.) (Velerianaceae)	Kyuragi, Saga City, Saga Pref., Japan	M. Tokuda	AB176718 ^§^	LC422105 ^¶^
*S.galiorum* (Kieffer)	*Galiummollugo* L. (Rubiaceae)	Wescot Downs, Surrey, UK	K.M. Harris	AB213410 ^#^	LC422108 ^¶^
*S.* (=*Asteralobia*) *humuli* (Shinji)	*Humulusjaponicus* Siebold & Zucc. (Cannabaceae)	Ogi City, Saga Pref., Japan	A.K. Elsayed	LC426397-LC426399	LC426420-LC426422
*S.paederiae* sp. n.	*Paederiafoetida* L. (Rubiaceae)	Nokubi, Nishinoomote City, Kagoshima Pref., Japan	K. Ogata	LC426400-LC426402	LC426423-LC426425
*S.* (=*Asteralobia*) *sasakii* (Monzen)	*Ilexcrenata* Thunberg (Aquifoliaceae)	Mount Daimonji, Kyoto Pref., Japan	N. Uechi	LC422071 ^¶^	LC422098 ^¶^
*S.* (=*Asteralobia*) *solidaginis* (Kovalev)	*Solidagopacifica* Juz. (Asteraceae)	Mount Litovka, Primorsky Territory, Russia	M. Tokuda et al.	LC426403	LC426426
*S.* (=*Asteralobia*) *soyogo* (Kikuti)	*Ilexpedunculosa* Miq. (Aquifoliaceae)	Mount Daimonji, Kyoto Pref., Japan	N. Uechi	LC422075 ^¶^	LC422102 ^¶^
*S.castanopsisae* sp. n.	*Castanopsissieboldii* (Makino) Hatus. (Fagaceae)	Hachijojima Island, Tokyo, Japan	T. Kikuchi	LC426404-LC426406	LC426427-LC426429
*S.usubai* sp. n.	*Trachelospermumasiaticum* (Sieb. et Zucc.) Nakai (Apocynaceae)	Mount Onigasawa, Nishino-omote, Nishino-omote City, Kagoshima Pref., Japan	K. Ogata	LC426407-LC426409	LC426430-LC426432

Sequences obtained from the Genbank: ^§^Tokuda et al. (2004); ^#^Tokuda et al. (2005); ^¶^Elsayed et al. in press.

The sequence data were analyzed with the maximum likelihood (ML) method using MEGA (ver. 6.0) (Tamura et al. 2013). Sequences of *Asphondyliagennadii* Marchal (AB198012 and AB115580) and *Pseudasphondylianeolitseae* Yukawa (AB334237 and LC422092) were used as outgroup species. The best model was identified with the jModelTest 2 software (Darriba et al. 2012, Guindon and Gascuel 2003) on the basis of hierarchical likelihood ratio tests (hLRT) was GTR+G+I.

## Results

### Taxonomy

#### Genus *Schizomyia* Kieffer, 1889

*Schizomyia* Kieffer, 1889: 183. Type species: *S.galiorum* Kieffer, 1889.

*Parasphondylia* Kieffer, 1913: 93. Type species: *P.variicornis* Kieffer, 1913.

*Asteralobia* Kovalev, 1964: 419. Type species: *A.doellingeriae* Kovalev, 1964. Syn. n.

*Euasteralobia* Kovalev, 1964: 430, as subg. of *Asteralobia*. Type species, *Asteralobiacalathidiphaga* Kovalev (mon.).

Kovalev (1964) erected the genus *Asteralobia*, all occurring in Russian Far East and Japan, based on the presence of constrictions on male flagellomeres. However, this character is observed in the type species of *Schizomyia*, *S.galiorum* (Fig. 69), and no other characters were found to differentiate between these genera. Therefore, the 12 species of *Asteralobia*, namely *A.asteris* Kovalev, *A.calathidiphaga* Kovalev, *A.clematidis* Fedotova, *A.doellingeriae* Kovalev, *A.humuli* Shinji, *A.kovalevi* Skuhravá, *A.patriniae* Shinji, *A.sasakii* Monzen, *A.soyogo* Kikuti, *A.solidaginis* Kovalev, *A.spiraeae* Fedotova and *A.veronicastrum* Fedotova, are combined here under *Schizomyia*.

*Schizomyia* is a cosmopolitan genus of 53 species, which are associated with over 30 host plants (Gagné and Jaschhof 2017; Elsayed et al. in press). With such a broad host range, *Schizomyia* is considered a catch-all genus defined only by plesiotypic characters and lack of synapomorphies (Gagné and Marohasy 1997; Gagné 1994). However, *Schizomyia* can be distinguished from the other genera of Schizomyiina by the following combination of characters: palpi four-segmented; tarsomeres I without ventroapical extension (Elsayed et al. in press), except *S.maricaensis* Sousa & Maia (Sousa and Maia 2007) and *S.novoguineensis* Kolesik (Kolesik and Butterill 2015); ovipositor protrusible, with needlelike protrusible portion; and larva usually with bilobed spatula and eight or fewer terminal papillae.

**Figures 1–4.**
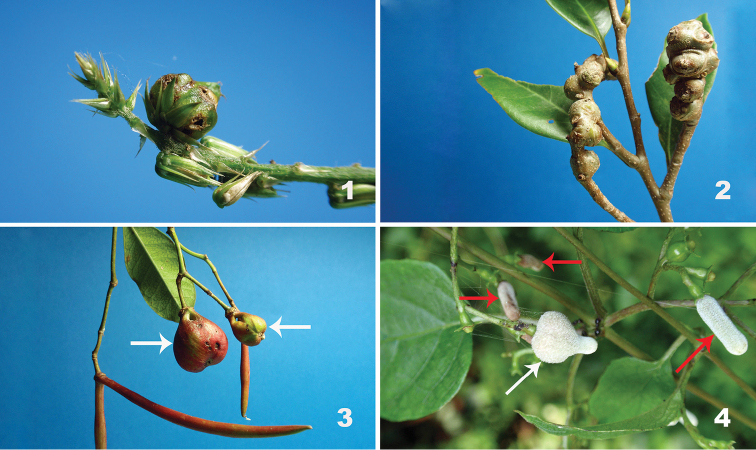
Galls of *Schizomyia* spp. **1** Fruit gall induced by *S.achyranthesae* on *Achyranthesbidentata* (Amaranthaceae) **2** Inflorescence galls induced by *S.castanopsisae* on *Castanopsissieboldii* (Fagaceae) **3** Fruit galls (arrows) induced by *S.usubai* on *Trachelospermumasiaticum* (Apocynaceae) **4** A flower bud gall (white arrow) induced by *S.paederiae* on *Paederiafoetida* (Rubiaceae) [red arrows indicate normal flower buds].

##### Schizomyia achyranthesae

Taxon classificationAnimaliaDipteraCecidomyiidae

Elsayed & Tokuda sp. n.

http://zoobank.org/AC909591-4D08-489E-AC19-9221F770F0D6

Figs 5–11 , 12–17; Table S1

###### Etymology.

The species name, *achyranthesae*, is based on the generic name the host plant, *Achyranthesbidentata* (Amaranthaceae).

###### Type material.

*Holotype*: 1♂ (KUEC): reared by A. K. Elsayed from a larva obtained from a fruit gall on *A.bidentata*, collected from Mount Tara, Saga Prefecture, Japan, on 7.x.2015, M. Tokuda leg., the larva departed from gall between 10–19.x.2015 and the adult male emerged on 3.ix.2016. *Paratypes*: All paratypes were reared from fruit galls on *A.bidentata* in Japan. 3 larvae: collected from Tokushima City, Tokushima Prefecture on 6.x.2001, M. Yukinari leg., larvae departed from galls on 12.x.2001; 5 larvae: collected from Tokushima City, Tokushima Prefecture on 30.x.2012, J. Yukawa et al. leg., larvae departed from galls on 30.x.2012; 8♀, 4 Pupal exuviae: collected from Takeo City, Saga Prefecture on 10.x.2015, A. Kita leg., larvae departed from galls between 13–19.x.2015, adults emerged on 1.ix.2016; 3♀: collected from Mount Hinokuma, Saga Prefecture on 16.x.2014, A. K. Elsayed & M. Tokuda leg., larvae departed from galls on 22.x.2014, adults emerged in summer 2015; 4 pupal exuviae: collected from Takeo City, Saga Prefecture on 10.x.2015, larvae departed from galls between 13–19.x.2015, adults emerged on 29.viii.2016; 10♂, 6♀: same data as holotype.

###### Description.

**Figures 5–11.**
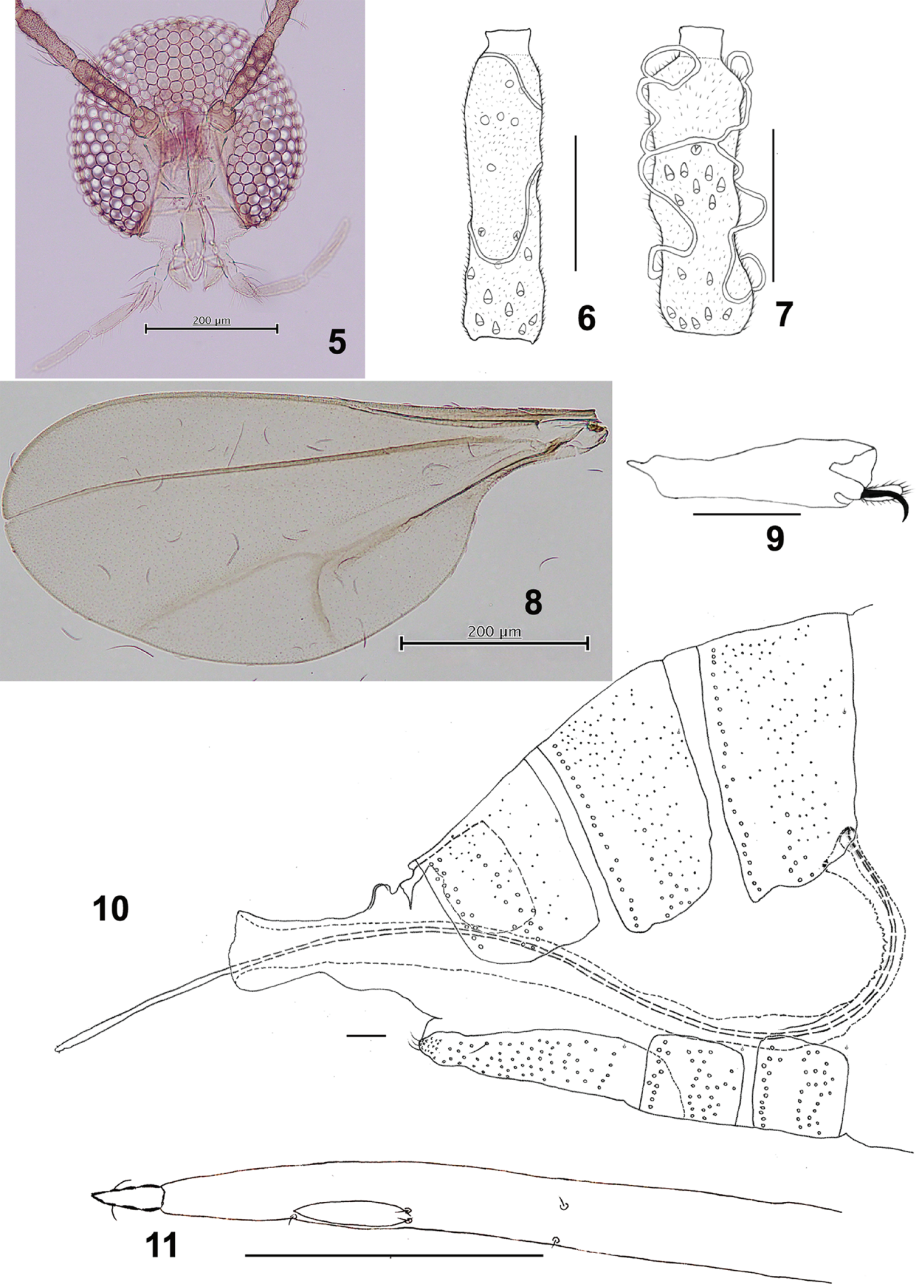
*Schizomyiaachyranthesae*. **5** Head **6** Ventral view of female antennal flagellomere V **7** Ventral view of male flagellomere V **8** Wing **9** Tarsomere V and acromere **10** Terminal part of female abdomen **11** Ovipositor apex. Scale bars: 50 µm (**6, 7, 9–11**), 200 µm (**5, 8**).

*Head* (Fig. 5): Compound eyes separated on vertex by a diameter of 0.0–0.75 facets, eye bridge 7–8 facets long, facets hexagonal. Fronto-clypeus with 15–21 setae (*n* = 6). Labrum and labella setose. Palpus 4-segmented: first segment ca 34 μm, second 1.3 times as long as the first, third 1.5 as long as the second, fourth 1.3 as long as the third. Antenna: scape slightly wider than long, pedicel rounded, flagellomeres I and II partially fused; female flagellomeres I–IX cylindrical with 2 connected rings of circumfila (Fig. 6); distal flagellomeres successively shorter; flagellomere X about as long as wide; flagellomere XI rounded, slightly wider than long; flagellomere XII rudimentary, partially fused with XI; male flagellomeres cylindrical with sinuous circumfila (Fig. 7).

*Thorax*: Wing (Fig. 8) length 1.60–2.02 mm (*n* = 6) in female, 1.43–1.73 mm (*n* = 5) in male: R_1_ join C before wing midlength, R_5_ join C just after wing apex, C broken after the conjunction with R_5_; wing fold present; M_3+4_ forked with Cu. Tarsal claws untoothed, bent after midlength; empodia shorter than claws, with long setulae apically; pulvilli not discernable (Fig. 9). Anepimeral setae 19–23 (*n* = 8); mesanepisternum scales 22–38 (*n* = 6); lateral scutal setae 21–28 (*n* = 8). Lengths of leg segments as in Suppl. material 1: Table S1.

*Female abdomen* (Figs 10, 11): Tergites with anterior pair of trichoid sensilla; tergites I–VII rectangular and evenly covered with scales, tergites I–VI with a posterior row of setae and some scattered setae laterally at midlength; tergite VII with 1–2 posterior rows of setae and some scattered setae laterally at midlength; tergite VIII bare, notched laterally, posterior margin with a pair of well-developed dorsal lobes. Sternites II–VI rectangular, bare and less pigmented medially, with the lateral pair of trichoid sensilla situated anterior to the sclerotized sternite, several scattered setae on the anterior half, and 1–2 rows of setae posteriorly; sternite VII about 2.6 times as long as preceding sternites, with anterior pair of trichoid sensilla laterally situated on the sternite and setae covering posterior two-thirds. Ovipositor: protrusible needlelike portion about 4 times as long as sternite VII (Fig. 10); cerci fused, each lobe with few setae (Fig. 11).

**Figures 12–17.**
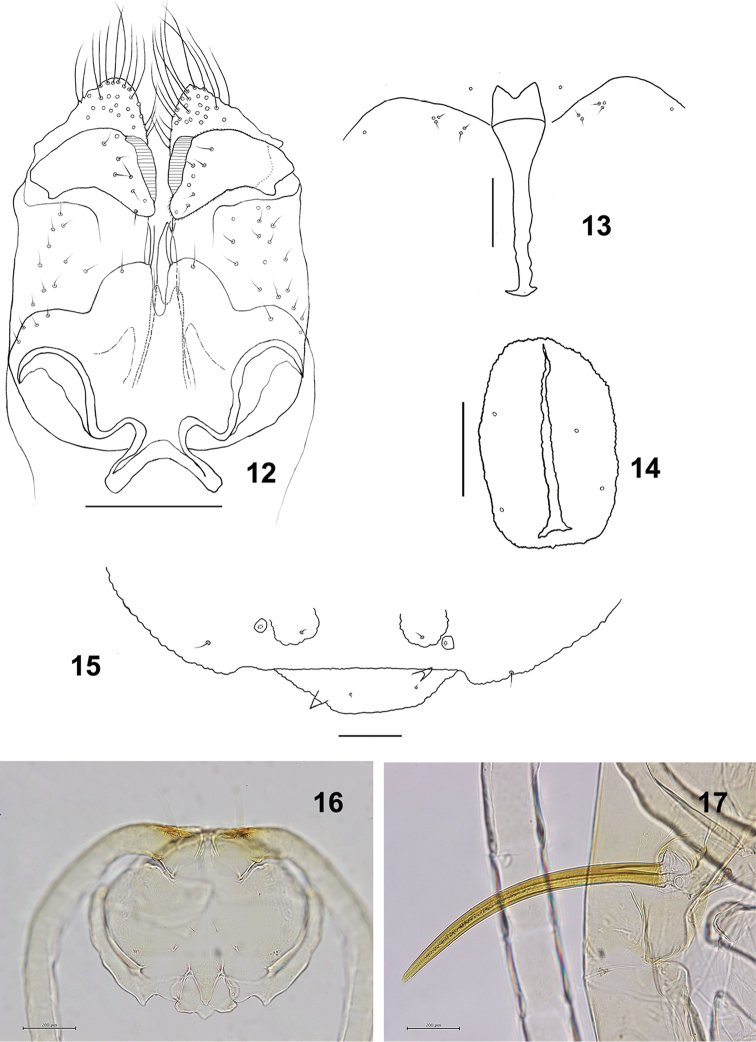
*Schizomyiaachyranthesae*. **12** Male terminalia **13** Larval spatula **14** Larval anus **15** Terminal larval segment dorsally **16** Ventral view of pupal head **17** Pupal prothoracic spiracle. Scale bars: 50 µm (**12–15**), 100 µm (**16, 17**).

*Male abdomen*: Tergites I–VII as in female; tergite VIII weakly sclerotized medially, with anterior pair of trichoid sensilla. Sternites II–VI as in female; sternite VII with lateral pair of trichoid sensilla situated anterior to the sclerotized sternite, several setae scattered anteriorly, median membranous bare area and 1–2 posterior row of setae; sternite VIII setose, with lateral pair of trichoid sensilla situated intersegmentally between sterna VII and VIII. Terminalia (Fig. 12): gonocoxite extending ventrally as convex lobe beyond of gonostylus; gonostylus stout, with unfused and compressed denticles, dorsally with setae on distal third, ventrally with a cluster of setae on the basal half. Cerci notched, each cercus with 4 strong setae and a few fine setae. Hypoproct shorter than cerci and aedeagus, bilobed, with a seta on each lobe. Parameres about half length of hypoproct. Aedeagus gradually tapering, acute apex, longer than cerci.

*Mature larva* (Figs 13–15): Cylindrical, yellow. Number and position of thoracic and abdominal spiracles as in other Asphondyliini (see Möhn 1961); 6 dorsal papillae present on all thoracic segments and abdominal segments I–VII, each with a seta; abdominal segment VIII with 2 dorsal lobes, each with a setose dorsal papilla. Sternal spatula bilobed (Fig. 13) with posterior portion about 3.6 times as wide as the base of the anterior free portion. Two groups of lateral papillae on all thoracic segments, the inner group of 2 setose papillae and the outer group of 2 setose and 1 asetose papillae. Two sternal papillae on each thoracic segment and 4 sternal papillae on abdominal segments I–VIII, all without setae and situated on slight swellings. Ventral papillae each with a seta on meso- and metathoracic segments and on abdominal segments I–VII. Anus situated ventrally, with simple opening and 4 asetose anal papillae (Fig. 14). Two pairs of pleural papillae present on all thoracic segments and abdominal segment VIII, and 3 pairs on abdominal segments I–VII. Terminal segment (Fig. 15) with 2 setose and 2 corniform terminal papillae.

*Pupa* (Figs 16, 17): Exuviae not pigmented except prothoracic spiracles and dorsal spines of abdomen. Antennal horns short; 2 pairs of cephalic papillae present, a pair with short seta; 2 pairs of lower facial papillae, a pair with seta; 3 lateral facial papillae present on each side, 1 with short seta, 2 without setae. Prothoracic spiracle, slightly curved, 23–29 μm long (*n* = 6), connected with trachea to the tip. Spiracles present on abdominal segments II–VI. Abdominal terga I–VIII each with anterior pair of trichoid sensilla and 2 pleural papillae; terga I–VII each with 3 pairs of dorsal papillae, only outermost pair with a seta; tergum VIII with a pair of dorsal papillae, each with a seta. Abdominal terga II–VIII each with 3–4 rows of spines on median third.

###### Distribution.

Japan: Honshu, Shikoku, Kyushu (Yukawa and Masuda 1996) and Tanegashima Island (Yukawa et al. 2013).

###### Gall and life history.

*Schizomyiaachyranthesae* induces subglobular fruit galls on *A.bidentata*, 5.07–5.17 mm in diameter (*n* = 5) (Fig. 1) [Gall No. C-246 in Yukawa and Masuda (1996)]. Based on Yukawa and Masuda (1996) and present observations, each gall contains 1–13 chambers and each chamber contains a single larva. The galls appear in September. The mature larvae leave the galls between October and November and overwinter in the soil. The adults of *S.achyranthesae* emerge during the flowering season of the host plant in August and September.

###### Remarks.

*Schizomyiaachyranthesae* is distinguishable from the known *Schizomyia* species, except *S.asteris* and *S.solidaginis*, by its shallowly constricted male flagellomeres, lateral position of anterior pair of trichoid sensilla and presence of four larval terminal papillae, as well as two setose papillae in inner group of lateral papillae. *S.achyranthesae* can be separated from *S.solidaginis* based on the larval characters as follows: *S.achyranthesae* possesses a more elongated sternal spatula than *S.solidaginis*; the inner group of lateral papillae consists of two setose papillae in *S.achyranthesae*, but one setose and one asetose papillae in *S.solidaginis*; the anal opening is simple in *S.achyranthesae*, while branched in *S.solidaginis*. Then, *S.achyranthesae* can be separated from *S.asteris* by the following features: female cerci is less divided in *S.achyranthesae*; dorsal setae are present on the gonostylus in *S.achyranthesae*, but absent in *A.asteris*; and the gonocoxite is only slightly extends ventrally beyond the gonostylus in *S.achyranthesae*, and the larval anal opening is simple in *S.achyranthesae* while branched in *S.asteris*.

##### Schizomyia diplocyclosae

Taxon classificationAnimaliaDipteraCecidomyiidae

Elsayed & Tokuda sp. n.

http://zoobank.org/C884D6A9-466D-45A8-9C63-A772B85E2539

Figs 18–24 , 25–29; Table S1

Characters given in *S.achyranthesae* except for the following:

###### Etymology.

The species name, *diplocyclosae*, is based on the generic name of the host plant, *Diplocyclospalmatus* (Cucurbitaceae).

###### Type material.

*Holotype*: 1♂ (KUEC): reared from a larva obtained from a flower bud gall on *D.palmatus*, collected from Hantagawa, Naha City, Okinawa Prefecture, Japan on 10.ii.2016, T. Ganaha-Kikumura leg., emerged on 14.iii.2016. *Paratypes*: All paratypes were reared from flower bud galls on *D.palmatus* in Japan. 4 larvae: collected from Gogayama, Nakijin Village, Okinawa Prefecture on 4.iii.2002, M. Tokuda leg., departed from galls on 9.iii.2002; 2 larvae: collected from Kinjo cho, Naha City, Okinawa Prefecture on 13.i.1977, S. Yamauchi leg.; 4 pupal exuviae: collected from Shuri, Naha City, Okinawa Prefecture, emerged in February 1977, S. Yamauchi leg.; 2 pupal exuviae, 3♂, 5♀: same data as holotype; 5 pupal exuviae, 3♂, 2♀: collected from Hantagawa, Naha City, Okinawa Prefecture on 10.ii.2016, T. Ganaha-Kikumura leg., emerged on 15.iii.2016; 1 pupal exuviae: collected from Hantagawa, Naha City, Okinawa Prefecture on 10.ii.2016, T. Ganaha-Kikumura leg., emerged on 16.iii.2016; 1 pupal exuviae: collected from Hantagawa, Naha City, Okinawa Prefecture on 10.ii.2016, T. Ganaha-Kikumura leg., emerged on 21.iii.2016.

###### Description.

**Figures 18–24.**
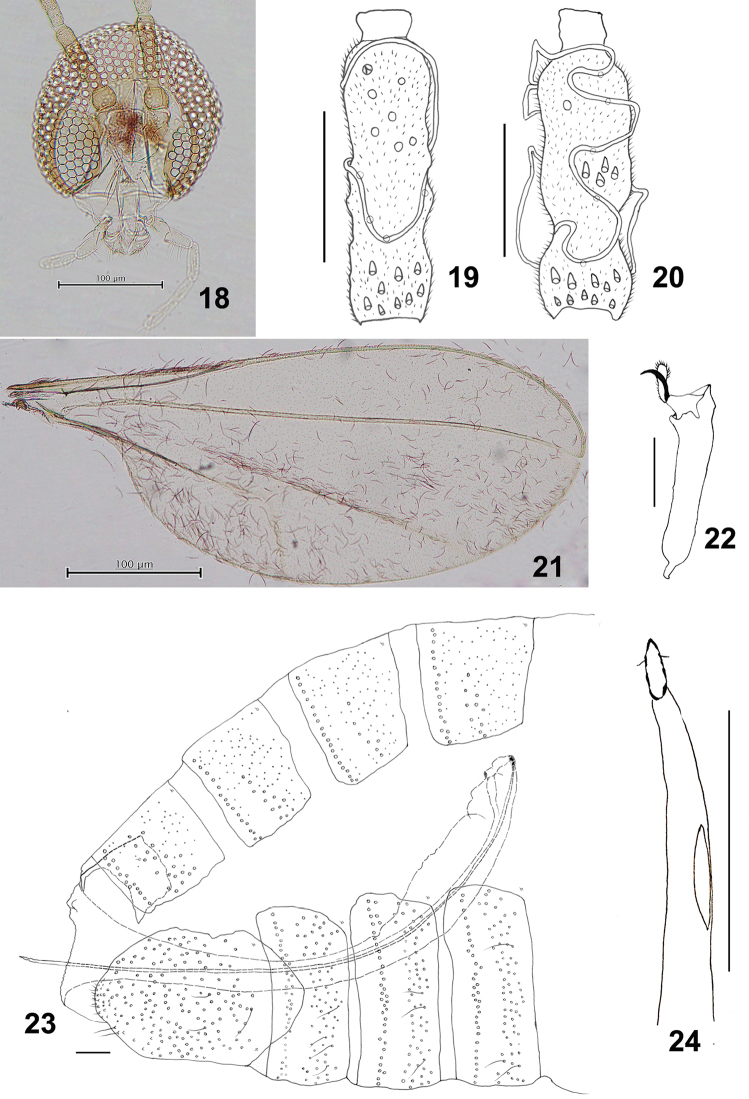
*Schizomyiadiplocyclosae*. **18** Head **19** Ventral view of female flagellomere V **20** Dorsal view of male flagellomere V **21** Wing **22** Tarsomere V and acromere **23** Terminal part of female abdomen **24** Ovipositor apex Scale bars: 100 µm (**18, 19**), 50 µm (**20–24**).

*Head* (Fig. 18): Fronto-clypeus with 17–24 setae (*n* = 6). Palpus: first segment ca 38.3 μm, second 1.4 times as long as the first, third 1.3 as long as the second, fourth 1.5 as long as the third.

*Thorax*: Wing (Fig. 21) length 2.15–2.26 mm (*n* = 5) in female, 1.70–2.12 (*n* = 5) in male. Empodia slightly longer than tarsal claws (Fig. 22). Anepimeral setae 11–18 (*n* = 8); mesanepisternum scales 20–38 (*n* = 8); lateral scutum setae 26–48 (*n* = 8). Lengths of leg segments as in Suppl. material 1: Table S1.

*Female abdomen* (Figs 23, 24): Posterior margin of tergite VIII with a pair of slightly developed dorsal lobes. Sternite VII about 2.5 times as long as preceding sternites. Ovipositor: distal protrusible needle-like portion about 3 times as long as sternite VII.

**Figures 25–29.**
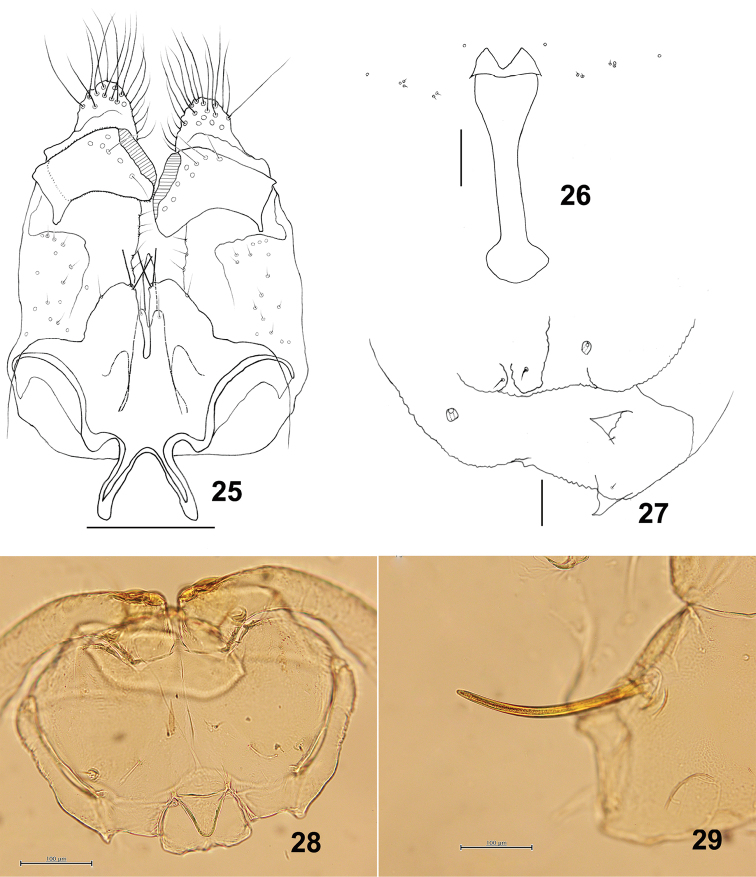
*Schizomyiadiplocyclosae*. **25** Male terminalia **26** Larval spatula **27** Terminal larval segments dorsally **28** Ventral view of pupal head **29** Prothoracic spiracle in pupa. Scale bars: 50 µm (**25–27**), 100 µm (**28, 29**).

*Male abdomen*: Terminalia (Fig. 25): Gonostylus dorsally with several setae on distal half.

*Mature larva*: Sternal spatula with posterior portion about 2.8 times as wide as the base of the anterior free portion (Fig. 26). Larval anus with 2 asetose anal papillae.

*Pupa* (Figs 28, 29): Prothoracic spiracle 280–310 μm long (*n* = 6).

###### Distribution.

Japan: Kikaijima Island and Okinawa-honto Island (Yukawa and Masuda 1996).

###### Gall and life history.

*Schizomyiadiplocyclosae* induces subglobular and pale green flower bud galls on *D.palmatus*, about 6–10 mm in diameter. Each gall consists of 10–45 chambers and each chamber contains a single larva [Gall No. C-409 in Yukawa and Masuda (1996)]. Galls become mature between December and March and larvae depart from galls to drop to the ground. The adults of *S.diplocyclosae* emerge in February and March when the larvae were reared under laboratory temperature (Yukawa and Masuda 1996, present data). Similar flower bud galls were found on *Melothrialiukiuensis* Nakai (Cucurbitaceae) and considered to be induced by this species or a closely related one (Yamauchi et al. 1982, Yukawa and Masuda 1996).

###### Remarks.

*Schizomyiadiplocyclosae* is morphologically very similar to *S.achyranthesae* but differs from it by the following characters: *S.diplocyclosae* has a shorter ovipositor (protrusible needle-like-portion three times as long as sternite VII while four times as long in *S.achyranthesae*), less developed dorsal lobes on the posterior margin of female tergite VIII, gonocoxite more pointed posteroapically, empodia longer than claws and larva with only two anal papillae (four in *S.achyranthesae*).

##### Schizomyia castanopsisae

Taxon classificationAnimaliaDipteraCecidomyiidae

Elsayed & Tokuda sp. n.

http://zoobank.org/30620DFD-56AD-4C7D-B1FE-035383F80721

Figs 30–36 , 37–42; Table S2

Characters as in *S.achyranthesae* except for the following:

###### Etymology.

The species name, *castanopsisae*, is based on the generic name of the host plant, *Castanopsissieboldii* (Fagaceae).

###### Type material.

*Holotype*: 1♂ (KUEC): reared from a larva obtained from an inflorescence gall on *C.sieboldii* by A. K. Elsayed, collected from Hachijojima Island on 6.xii.2014, T. Kikuchi leg., emerged on 24.ii.2015. *Paratypes*: All paratypes were reared by A. K. Elsayed from inflorescence galls on *C.sieboldii* in Japan. 2 larvae: collected from Hachijojima Island on 6.xii.2014, T. Kikuchi leg., departed from galls on 22.xii.2014; 5 larvae: collected from Hachijojima Island on 6.xii.2014, T. Kikuchi leg., departed from galls on 25.xii.2014; 6 pupal exuviae, 2♀, 3♂: collected from Shikinejima Island on 10.xii.2014, M. Tokuda leg., emerged between 24.i–20.ii.2015; 3 pupal exuviae, 4♀, 2♂: collected from Hachijojima Island on 6.xii.2014, T. Kikuchi leg., emerged between 20.ii–5.iii.2015.

###### Description.

**Figures 30–36.**
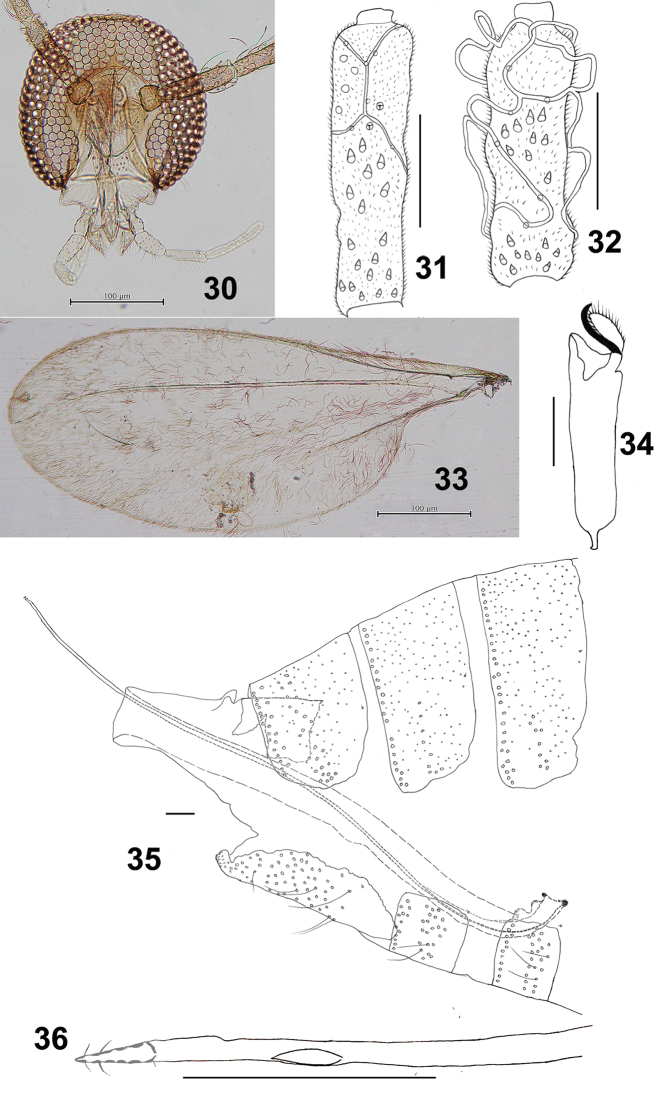
*Schizomyiacastanopsisae*. **30** Head **31** Ventral view of female flagellomere V **32** Ventral view of male flagellomere V **33** Wing **34** Tarsomere V and acromere **35** Terminal part of female abdomen **36** Ovipositor apex. Scale bars: 50 µm (**31, 32, 34–36**), 100 µm (**30, 33**).

*Head* (Fig. 30): Fronto-clypeus with 10–16 setae (*n* = 6). Palpus: first segment ca 43 μm, second 1.4 times as long as the first, third 1.5 as long as the second, fourth 1.2 as long as the third.

*Thorax*: Wing (Fig. 33) length 2.04–2.74 mm (*n* = 6) in female, 2.04–2.56 mm (*n* = 4) in male. Anepimeral setae 8–15 (*n* = 8); mesanepisternum scales 15–26 (*n* = 7); lateral scutum setae 15–27 (*n* = 8). Lengths of leg segments as in Suppl. material 1: Table S2.

*Female abdomen* (Figs 35, 36): Sternite VII about 3 times as long as preceding. Ovipositor: protrusible needle-like portion about 3.3 as long as sternite VII.

**Figures 37–42.**
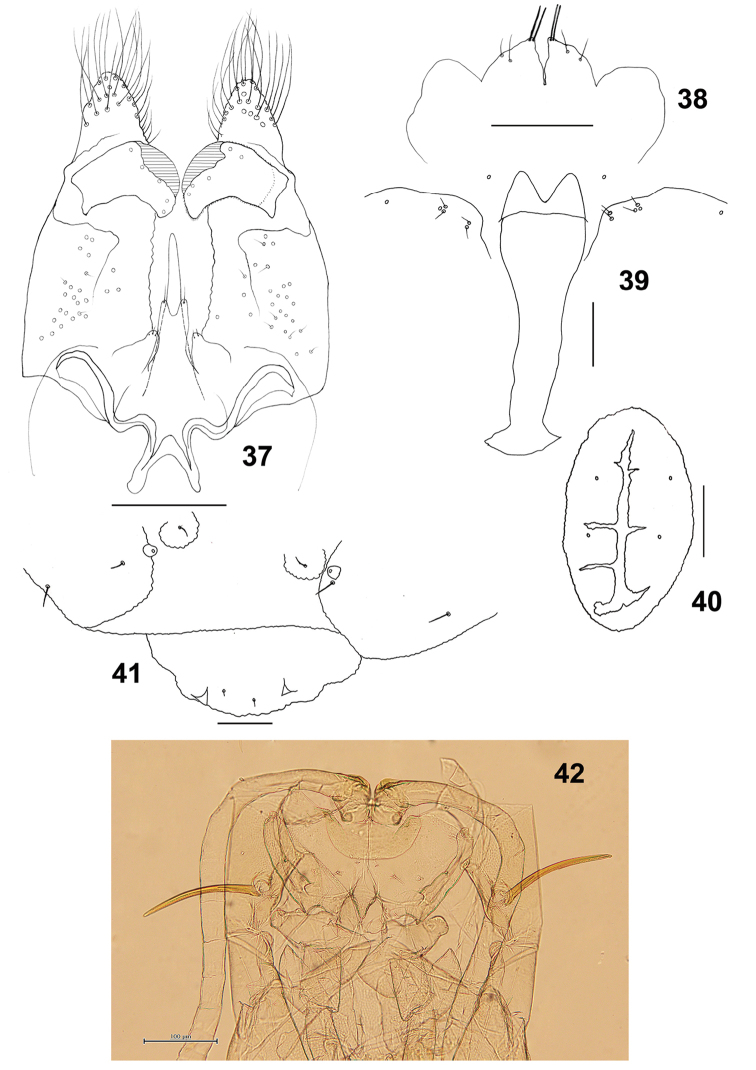
*Schizomyiacastanopsisae*. **37** Male terminalia **38** Male cerci **39** Larval spatula **40** Larval anus **41** Terminal larval segments dorsally **42** Ventral view of pupal head and thorax. Scale bars: 50 µm (**37–41**), 100 µm (**42**).

*Male abdomen*: Terminalia (Figs 37, 38): Gonocoxite length about 3.3 times as long as gonostylus.

*Mature larva*: Sternal spatula (Fig. 39) with posterior portion about 2.8 times as wide as the base of the anterior free portion. Anus opening branched (Fig. 40).

*Pupa* (Fig. 42): Prothoracic spiracle 280–330 μm long (*n* = 6).

###### Distribution.

Japan: The Izu Islands (from Niijima to Aogashima) (Tokuda et al. 2012a, b, 2013, 2015, Tokuda and Kawauchi 2013a), Kyushu (Kagoshima and Miyazaki Prefectures) (Nagai 2010, Tokuda and Kawauchi 2013b), Tanegashima Island (Yukawa et al. 2013) and Okinawa-honto Island (Yamauchi et al. 1982).

###### Gall and life history.

*Castanopsissieboldii* inflorescences galled by *S.castanopsisae* are rather irregularly swollen, 5.7–15.7 mm in diameter and 6.2–30.9 mm in length (Fig. 2) [Gall No. C-163 in Yukawa and Masuda (1996)]. Each gall consists of up to 30 chambers and each chamber contains a single larva. Mature larvae of *S.castanopsisae* left the galls collected in the Izu Islands in December within few days after the collection. Larvae were kept with soil in the laboratory and adults emerged the following January, February and March.

###### Remarks.

*Schizomyiacastanopsisae* is morphologically close to *S.asteris*, *S.achyranthesae* and *S.diplocyclosae*. *Schizomyiacastanopsisae* can be separated from *S.asteris* by a shorter ovipositor (protrusible needle-like portion 3.3 times as long as sternite VII, while 5.7 times in *S.asteris*), the presence of dorsal setae on the gonostyli, and the tooth of gonostylus, which extends more dorsally than in that of *S.asteris*; from *S.achyranthesae* by a shorter ovipositor (four times as long as sternite VII in *S.achyranthesae*), more posteroapically pointed gonocoxite, and branched anal opening of larva; and from *S.diplocyclosae* by shorter empodia than tarsal claws (empodia are as long as claws in *S.diplocyclosae*) and the number of larval anal papillae (four in *S.castanopsisae* while two in *S.diplocyclosae*).

##### Schizomyia usubai

Taxon classificationAnimaliaDipteraCecidomyiidae

Elsayed & Tokuda sp. n.

http://zoobank.org/ACDD6BD7-7BAD-4330-8A79-03066DACA3F1

Figs 43–49 , 50–55; Table S2

Characters as in *S.achyranthesae* except for the following:

###### Etymology.

The species name, *usubai*, honors the late Mr Shigeshi Usuba who reared adults of this species for the first time.

###### Type material.

*Holotype*: 1♂ (KUEC): reared by A. K. Elsayed from a larva obtained from a fruit gall on *T.asiaticum*, collected from Torinosu, Tanabe City, Wakayama Prefecture, Japan, I. Matoba leg., emerged on 22.v.2017. *Paratypes*: All paratypes were reared from fruit galls on *T.asiaticum* in Japan. 4 larvae: collected from Mount Takakuma, Kagoshima Prefecture in 1969, J. Yukawa leg.; 4 larvae: galls collected from Imuta Lake-side, Kedouin, Satsuma-sendai City, Kagoshima Prefecture on 2.xi.1978, S. Sako leg.; 4 pupal exuviae, 2♂, 2♀: collected from Torinosu, Tanabe City, Wakayama Prefecture, I. Matoba leg., reared by A. K. Elsayed, emerged on 18.v.2017; 2 pupal exuviae, 1♀, 2♂: same data as holotype.

###### Description.

**Figures 43–49.**
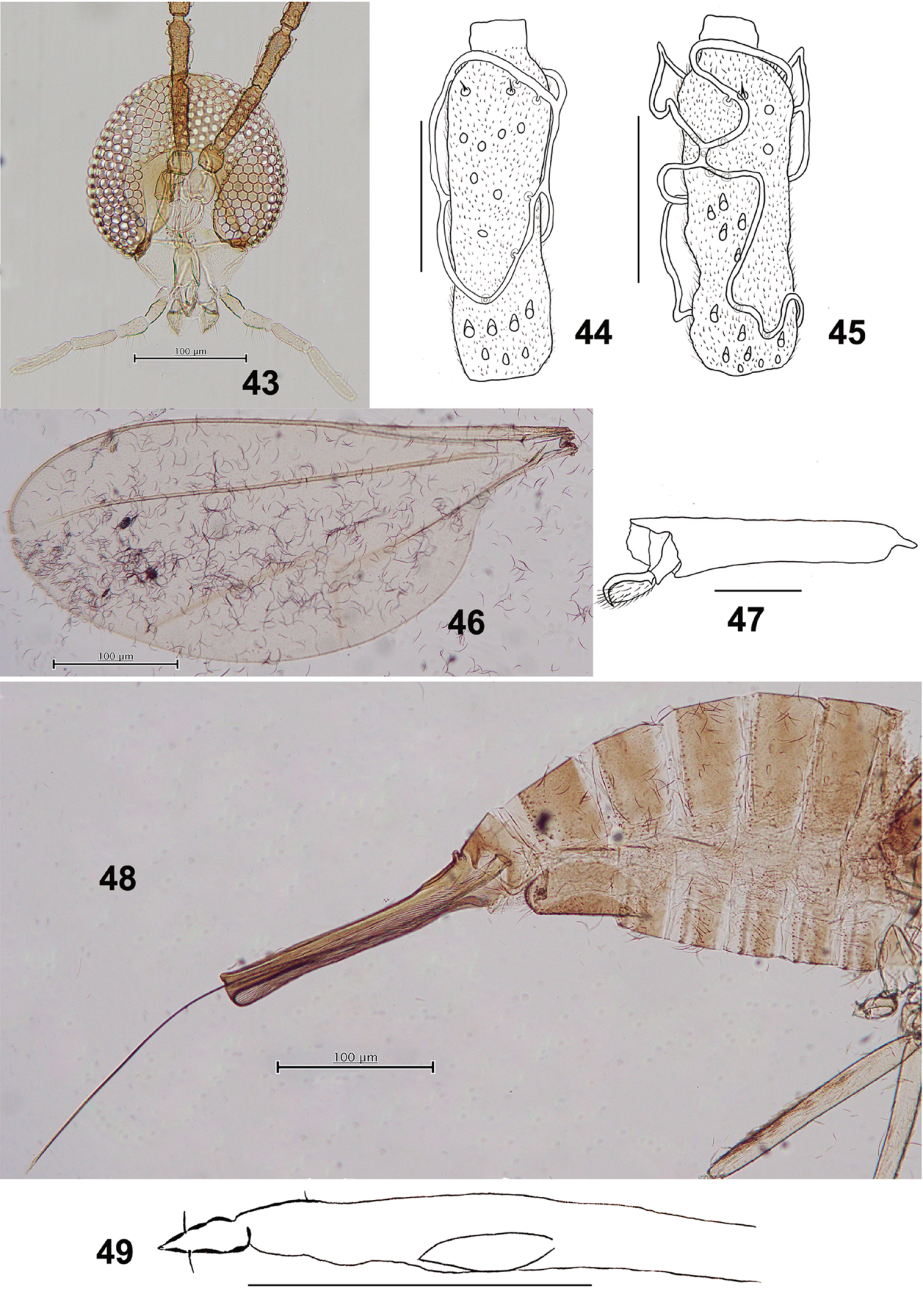
*Schizomyiausubai*. **43** Head **44** Ventral view of female flagellomere V **45** Ventral view of male flagellomere V **46** Wing **47** Tarsomere V and acromere **48** Terminal part of female abdomen **49** Ovipositor apex. Scale bars: 50 µm (**44, 45, 47, 49**), 100 µm (**43, 46, 48**).

*Head* (Fig. 43): Compound eyes separated on vertex by a diameter of 0.0–0.5 facets. Fronto-clypeal setae 15–16 setae (*n* = 4). Palpus: first segment ca 53.5 μm, second about as long as the first, third 1.6 as long as the second, fourth 1.4 as long as the third.

*Thorax*: Wing (Fig. 46) length 2.03–2.34 mm (*n* = 4) in female, 1.80–1.95 mm (*n* = 3) in male. Empodia as long as claws (Fig. 47). Anepimeral setae 10–20 (*n* = 4); mesanepisternum scales 17–40 (*n* = 4); lateral scutum setae 19–27 (*n* = 4). Lengths of leg segments as in Suppl. material 1: Table S2.

*Female abdomen* (Figs 48, 49): Sternite VII about 2.6 times as long as preceding. Ovipositor: protrusible needle-like portion about 4.5 as long as sternite VII.

**Figure 50–55.**
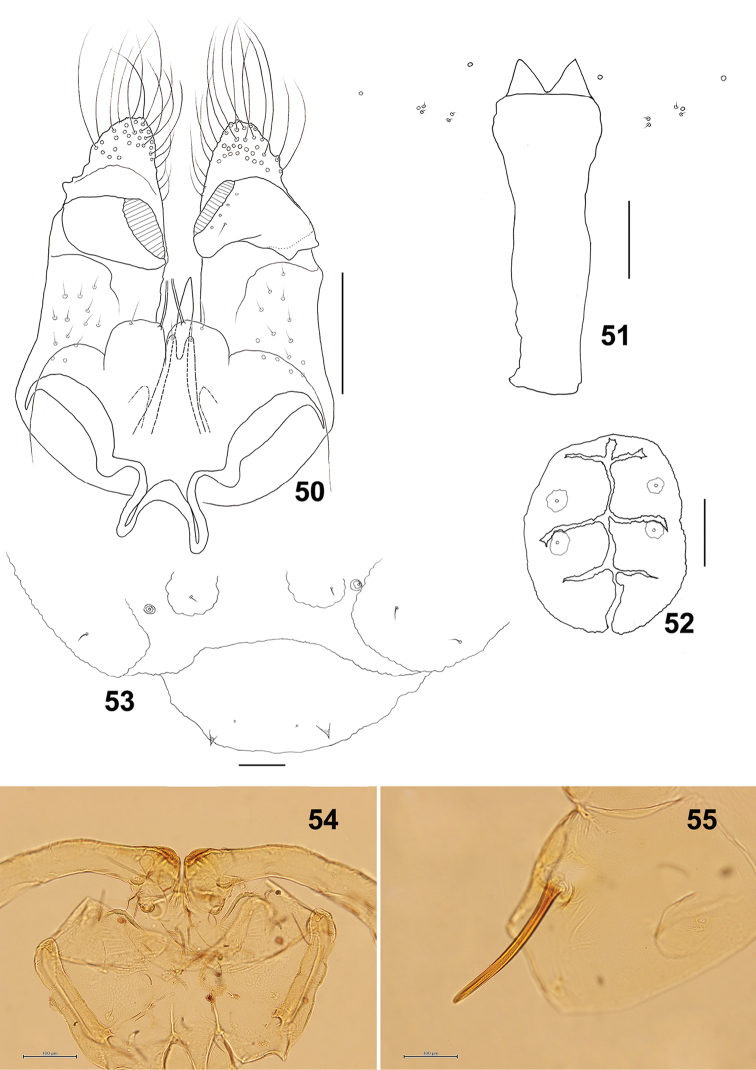
*Schizomyiausubai*. **50** Male terminalia **51** Larval spatula **52** Larval anus **53** Terminal larval segments dorsally **54** Ventral view of pupal head **55** Pupal prothoracic spiracle. Scale bars: 50 µm (**50–53**), 100 µm (**54, 55**).

*Male abdomen*: Terminalia (Fig. 50): Gonocoxite with developed, pointed apical lobe extending beyond gonostylus.

*Mature larva*: Sternal spatula (Fig. 51) with posterior portion about 3.5 times as wide as the base of the anterior free portion. Anus with branched opening (Fig. 52).

*Pupa* (Figs 54, 55): Prothoracic spiracle 250–350 μm long (*n* = 6).

###### Distribution.

Japan: The Izu Islands (Tokuda et al. 2012b, 2013, Tokuda and Kawauchi 2013) Honshu, and Kyushu (Yukawa and Masuda 1996).

###### Gall and life history.

The normal fruit of *Trachelospermumasiaticum* (Apocynaceae) is V-shaped, consisting of a pair of very long and thin seed pods. When the fruits are galled by *S.usubai*, the apical parts of the fruit become fused and swollen, more or less cat-bell shaped (Fig. 3), about 12–18 mm in diameter and 27 mm in length [Gall No. D-033 in Yukawa and Masuda (1996)]. Each gall consists of 10–25 chambers and each chamber contains 10–25 larvae. Galls mature between late September and October and the larvae depart from galls to overwinter in soil. The adults of *S.usubai* emerge between late April and July (Yukawa 1978; Yukawa and Masuda 1996; present study). Similar galls probably induced by this species were found on TrachelospermumgracilipesHook. f.var.kiukiuense (Hatus.) Kitam. on Tanegashima Island (Yukawa et al. 2013).

###### Remarks.

*Schizomyiausubai* is close to *S.asteris*, *S.achyranthesae*, *S.diplocyclosae* and *S.castanopsisae*. *Schizomyiausubai* can be distinguished from *S.asteris* by a shorter ovipositor (protrusible needle-like portion about 4.5 times as long as sternite VII, while 5.5 times in *S.asteris*), longer empodia, and the presence of dorsal setae on gonostyli; from *S.achyranthesae* and *S.diplocyclosae* by a longer ovipositor (four and three times as long as sternite VII in *S.achyranthesae* and *S.diplocyclosae*, respectively), longer empodia, and branched opening of the larval anus. In addition, larva of *S.usubai* has four anal papillae, but two in *S.diplocyclosae*. *Schizomyiacastanopsisae* is very similar to *S.usubai*, but can be separated by a shorter ovipositor (protrusible needle-like portion about 3.3 times as long as sternite VII, while 4.5 times in *S.usubai*), longer empodia, and less compressed circumfila of female flagellomeres.

##### Schizomyia paederiae

Taxon classificationAnimaliaDipteraCecidomyiidae

Elsayed & Tokuda sp. n.

http://zoobank.org/DE35F88A-484D-45CE-9BC2-4356E0A1DE97

Figs 56–62 , 63–67; Tables S3

Characters as in *S.achyranthesae* except for the following:

###### Etymology.

The species name, *paederiae*, is based on the generic name of the host plant, *Paederiafoetida* (Rubiaceae).

###### Type material.

*Holotype*: 1♂ (KUEC): reared from a larva obtained from a flower bud gall on *P.foetida*, collected from Misawa, Ogori City, Fukuoka Prefecture, Japan, K. Matsunaga leg., emerged between 11–15.viii.2017. *Paratypes*: All paratypes were reared from flower bud galls on *P.foetida* in Japan. 11 larvae: collected from Nishinoomote, Nishinoomote City, Kagoshima Prefecture, on 24.ix.2014, K. Ogata leg.; 4 pupal exuviae, 2♂, 7♀: same data as holotype.

###### Description.

**Figures 56–62.**
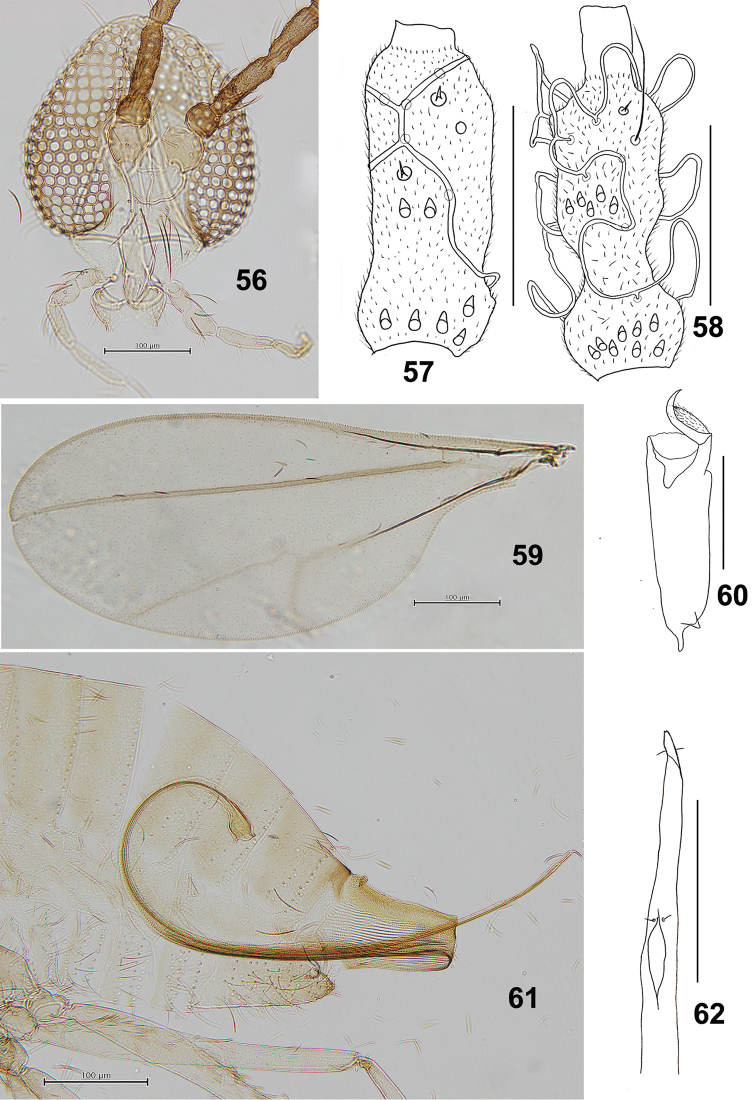
*Schizomyiapaederiae*. **56** Head **57** Dorsal view of female flagellomere V **58** Dorsal view of female flagellomere V **59** Wing **60** Tarsomere V and acromere **61** Terminal part of female abdomen **62** Ovipositor apex. Scale bars: 50 µm (**57, 58, 60, 62**), 100 µm (**56, 59, 61**).

*Head* (Fig. 56): Compound eyes separated on vertex by a diameter of 0.0–1.5 facets, eye bridge consist of 6–7 facets long. Fronto-clypeus with 11–13 setae (*n* = 4). Palpus: first segment ca 28.6 μm, second 1.3 times as long as the first, third 1.4 as long as the second, fourth 1.2 as long as the third.Male flagellomeres with deep basal constriction and elongated necks (Fig. 58).

*Thorax*: Wing (Fig. 59) length 1.16–1.57 mm (*n* = 5) in female, 1.04–1.36 mm (*n* = 3) in male. Anepimeral setae 9 or 10 (*n* = 5); mesanepisternum scales 5–10 (*n* = 6); lateral scutum setae 23–28 (*n* = 5). Empodia as long as tarsal claws (Fig. 60). Lengths of leg segments as in Suppl. material 1: Table S3.

*Female abdomen* (Figs 61, 62): Anterior pair of trichoid sensilla situated medially on abdominal sternites II–VI; sternite VII about 3.4 times as long as preceding sternites. Ovipositor: protrusible needle-like portion about 4.8 times as long as sternite VII.

**Figures 63–67.**
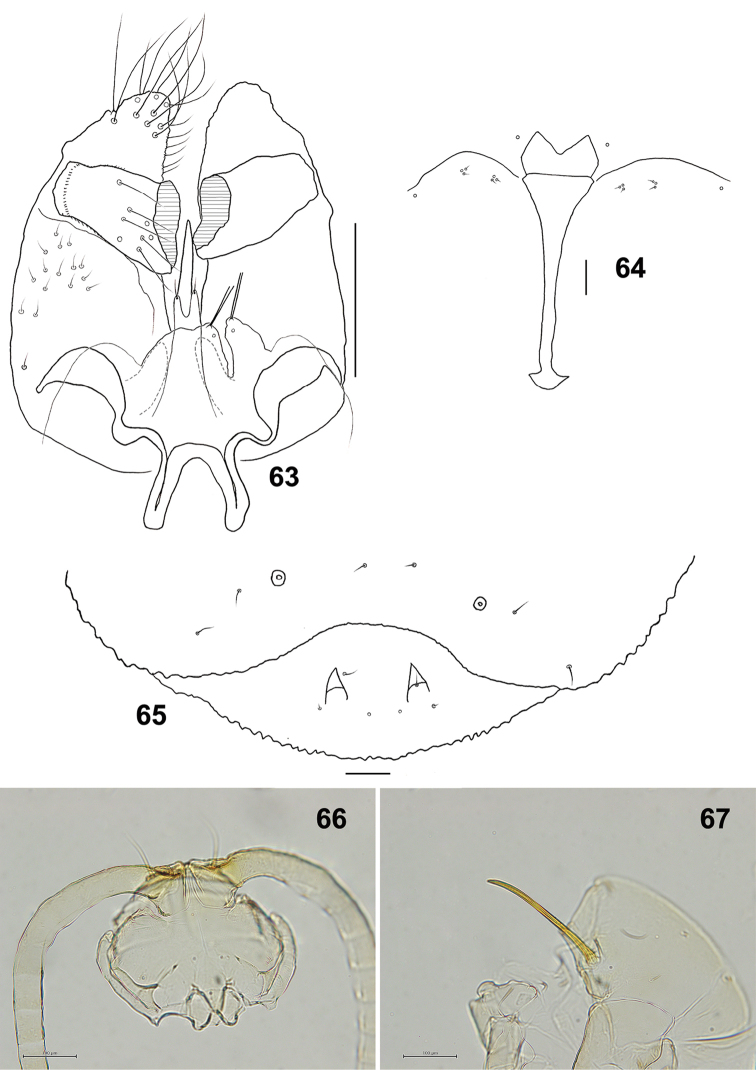
*Schizomyiapaederiae*. **63** Male terminalia **64** Larval spatula **65** Terminal larval segments dorsally **66** Ventral view of pupal head **67** Pupal prothoracic spiracle. Scale bars: 50 µm (**63–65**), 100 µm (**66, 67**).

*Male abdomen*: Anterior pair of trichoid sensilla situated medially on sternites II–VI and laterally on sternite VIII, sternite VIII with scattered setae. Terminalia (Fig. 63): Gonostylus dorsally with several setae on distal half, with unfused and compressed denticles.

*Mature larva*: Abdominal segment VIII with 2 setose dorsal papillae. Posterior portion of sternal spatula about 3.3 times as wide as the base of the anterior free portion (Fig. 64); 2 groups of lateral papillae present on all thoracic segments, each consisting of 2 setose and 1 asetose papillae. Terminal segment with 8 terminal papillae, consisting of 4 setose, 2 asetose and 2 corniform ones (Fig. 65).

*Pupa* (Figs 66, 67): Prothoracic spiracle 230–290 μm long (*n* = 4).

###### Distribution.

Japan: Honshu, Shikoku, Kyushu, and Yakushima Island (Yukawa and Masuda 1996).

###### Gall and life history.

*Schizomyiapaederiae* induces flower bud galls on *P.foetida*. Basal parts of the galled flower buds are swollen, 3.0–5.6 mm in diameter and 4.0–6.1 mm in length (Fig. 4) [Gall No. D-037 in Yukawa and Masuda (1996)]. Galls are single-chambered and each gall contains 1–10 larvae. The larvae depart from mature galls from late August to September and overwinter in the soil. The adults of *S.paederiae* emerge in early August when the flower buds are available on the host plant (Yukawa and Masuda 1996).

###### Remarks.

*Schizomyiapaederiae* is distinguishable from other *Schizomyia* species, except four Russian species, i.e. *S.calathidiphaga*, *S.clematidis*, *S.spiraeae*, and *S.veronicastrum*, by its deeply constricted male flagellomeres (Kovalev 1964; Fedotova 2002). Firstly, the adults of *S.paederiae* differs from *S.calathidiphaga* by a slightly longer ovipositor (protrusible needle-like portion about 4.8 times as long as sternite VII, while 4.5 times in *S.calathidiphaga*), longer empodia (empodia are as long as claws in *S.paederiae*, but shorter in *S.calathidiphaga*), the position of gonostylus tooth (mostly covering only the apical margin in *S.paederiae*, but on the posteroapical margin in *S.calathidiphaga*), and the arrangement of papillae on the larval terminal segment (the two asetose terminal papillae are situated more posteriorly in *S.paederiae*, while more anteriorly in *S.clathidiphaga*). Then, the adults of *S.paederiae* can be separated from *S.clematidis*, *S.spiraeae* and *S.veronicastrum* by a longer neck of male flagellomere III, which is about 0.25 as long as node in *S.paederiae* but about 0.15 as long as node in other species, the position of gonostylus tooth (mostly covering the apical margin in *S.paederiae*, but on the posteroapical margin in the other species), and a much narrower hypoproct than *S.clematidis*.

##### Schizomyia galiorum

Taxon classificationAnimaliaDipteraCecidomyiidae

Kieffer, 1889

Figs 68–73 , 74–76; Table S3

Characters as in *S.achyranthesae* except for the following:

###### Material examined.

2♂, 3♀ (Mamaev collection: slide no. B1-251369): collected from Rybatskij, Lithuania on 19.vii.1969; 1♀, 1 pupal exuviae (J. J. Kieffer’s specimen in Felt collection).

###### Description.

*Head*: Compound eyes with rounded facets; facets on vertex and eye bridge unobservable because the specimens mounted laterally. Palpus: first segment ca 23.4 μm, second 1.6 times as long as the first, third 1.4 as long as the second, fourth 1.4 as long as the third.

**Figures 68–73.**
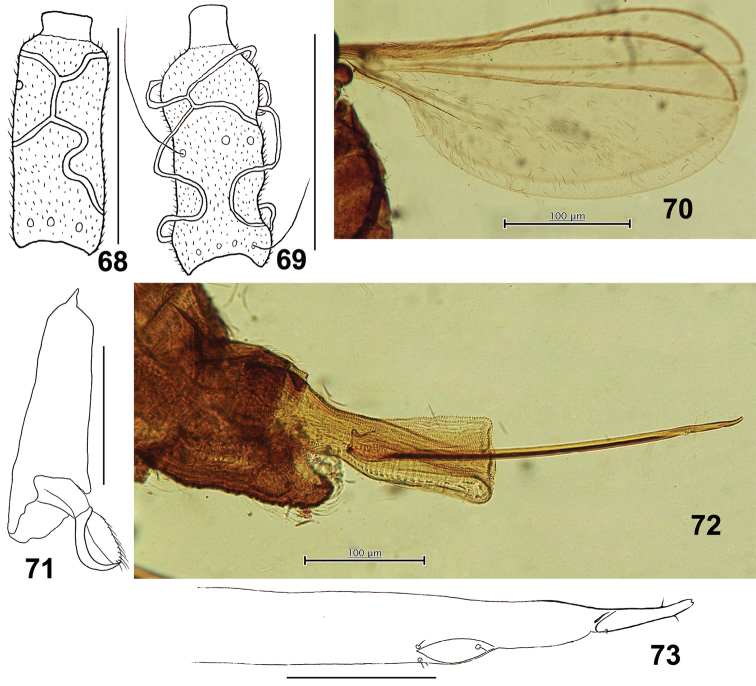
*Schizomyiagaliorum*. **68** Lateral view of female flagellomere V **69** Lateral view of male flagellomere V **70** Wing **71** Tarsomere V and acromere **72** Terminal part of female abdomen showing the ovipositor **73** Ovipositor apex. Scale bars: 50 µm (**68, 69, 71, 73**), 100 µm (**70, 72**).

*Thorax*: Wing (Fig. 70) length 1.33–1.55 mm (*n* = 2) in male, 1.93 mm (*n* = 1) in female; R_5_ joining C just before wing apex. Empodia as long as claws (Fig. 71). Anepimeral setae 9–15 (*n* = 5); mesanepisternum scales 14–18 (*n* = 4); lateral scutum setae 17–37 (*n* = 5). Lengths of leg segments as in Suppl. material 1: Table S3.

*Female abdomen*: Posterior margin of tergite VIII without dorsal lobes. Sternites with median pair of trichoid sensilla. Sternite VII about twice as long as VI. Ovipositor (Figs 72, 73): protrusible needle-like portion short, about 1.9 times as long as sternite VII; cerci fused, with few fine setae (Fig. 73).

**Figure 74–76.**
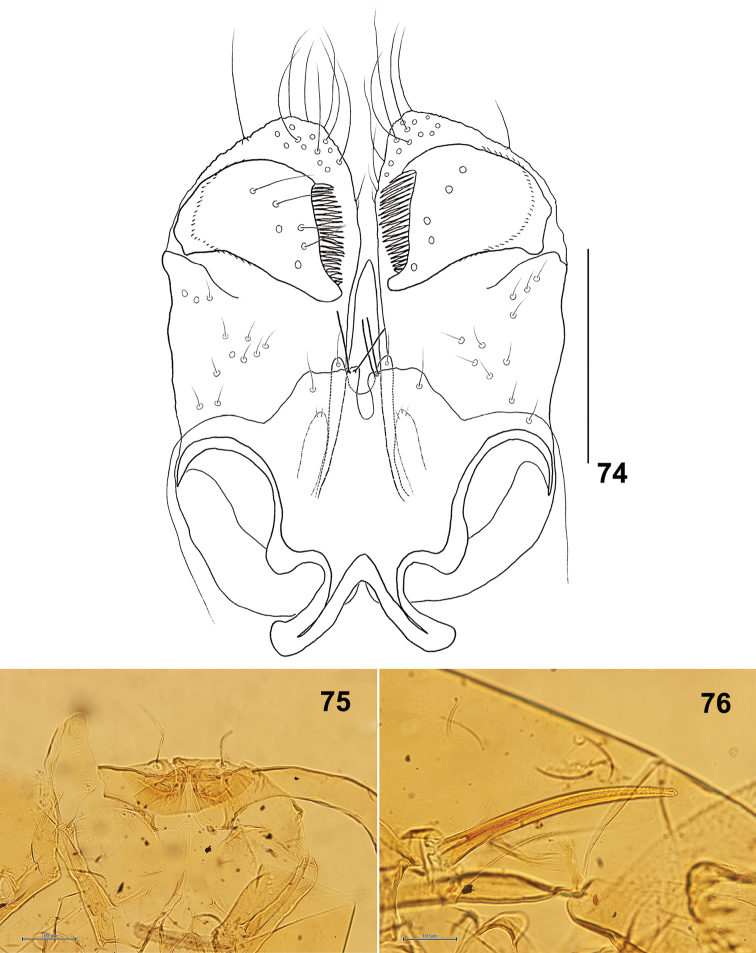
*Schizomyiagaliorum*. **74** Male terminalia **75** Ventral view of pupal head **76** Pupal prothoracic spiracle. Scale bars: 50 µm (**74**), 100 µm (**75, 76**).

*Male abdomen*: Sternites with median pair of trichoid sensilla. Sternite VII with two posterior rows of setae. Terminalia (Fig. 74): Gonocoxite with slightly developed apical lobe. Gonostylus with several setae on distal half dorsally, with group of setae on the basal half ventrally, and with distinctive unfused denticles. Hypoproct slightly longer than cerci.

*Mature larva*: Sternal spatula bilobed, the anterior free portion slightly wider than the posterior portion (Kieffer 1889). Two groups of lateral papillae present on each side of the spatula, each group of 2 setose and 1 asetose papillae. Terminal segment with 6 setose and 2 corniform terminal papillae (Möhn 1955).

*Pupa* (Figs 75, 76): Antennal horns slightly developed. Prothoracic spiracle 240 μm long (*n* = 1).

###### Distribution.

Widespread Europe, Algeria and Kazakhstan (Gagné and Jaschhof 2017).

###### Remarks.

This species is distinguished from eastern Holarctic congeners by the distinctly short ovipositor, the absence of dorsal lobes on the posterior margin of female tergite VIII, and the conjunction of wing vein C with R_5_ before wing apex.

##### Schizomyia patriniae

Taxon classificationAnimaliaDipteraCecidomyiidae

Shinji, 1938 comb. rev.

Figs 77–83 , 84–85; Table S4

Schizomyia patriniae Shinji, 1938: 372.
Asphondylia partriniae Shinji, 1944: 376, missp. of patriniae.
Asteralobia patriniae (Shinji, 1938)

Characters as in *S.achyranthesae* except for the following:

###### Material examined.

All obtained from flower bud galls on *Patriniavillosa* (Valerianaceae) in Japan. 6 larvae: collected from Maruyama, Ojiya City, Niigata Prefecture on 12.x.1981, K. Yamagishi leg. 1♂: Iozan, Kanazawa City, Ishikawa Prefecture on 17.x.1978, emerged on 22.iv.1979, J. Yukawa leg. 2♂, 4♀, 2 pupal exuviae: collected from Kyuragi, Karatsu City, Saga Prefecture on 12.x.2015, M. Tokuda leg., emerged on 23.viii.2016, reared by A. K. Elsayed. 2♂, 3♀, 2 pupal exuviae: collected from Kyuragi, Karatsu City, Saga Prefecture on 12.x.2015, M. Tokuda leg., emerged on 28.viii.2016, reared by A. K. Elsayed. 2♂: collected from Kyuragi, Karatsu City, Saga Prefecture on 12.x.2015, M. Tokuda leg., emerged on 29.viii.2016, reared by A. K. Elsayed.

###### Description.

**Figures 77–83.**
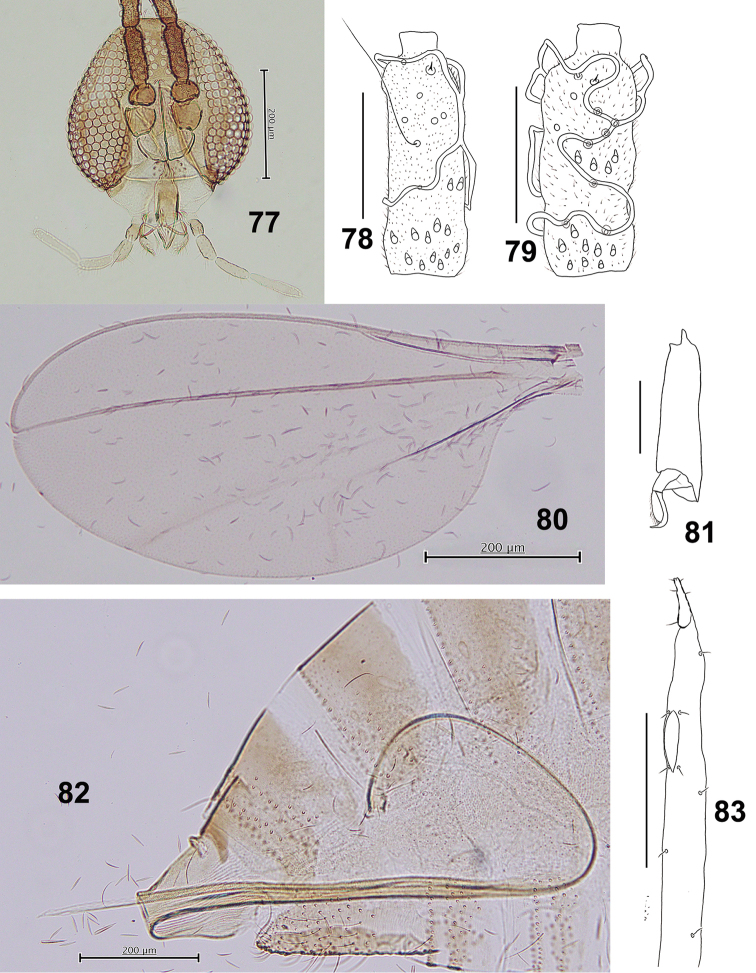
*Schizomyiapatriniae*. **77** Head **78** Dorsal view of female flagellomere V **79** Ventral view of female flagellomere V **80** Wing **81** Tarsomere V and acromere **82** Terminal part of female abdomen **83** Ovipositor apex. Scale bars: 50 µm (**78, 79, 81, 83**), 200 µm (**77, 80, 82**).

*Head* (Fig. 77): Compound eyes separated on vertex by a diameter of 0.25–1 facets, eye bridge consist of 5–6 facets long. Fronto-clypeus with 15–20 setae (*n* = 9). Palpus: first segment ca 34.1 μm, second 1.4 times as long as the first, third 1.4 as long as the second, fourth segment 1.5 as long as the third.

*Thorax*: Wing (Fig. 80) length 1.83–2.09 mm (*n* = 5) in female, 1.50–1.83 mm (*n* = 5) in male. Anepimeral setae 17–23 (*n* = 8); mesanepisternum scales 16–20 (*n* = 7); lateral scutum setae 32–46 (*n* = 8). Lengths of leg segments as Suppl. material 1: Table S4.

*Female abdomen* (Figs 82, 83): Median pair of trichoid sensilla present on sternites II–VII. Sternite VII about 3 times as long as preceding. Ovipositor: protrusible needle-like portion about 4.8 as long as sternite VII.

**Figures 84–85.**
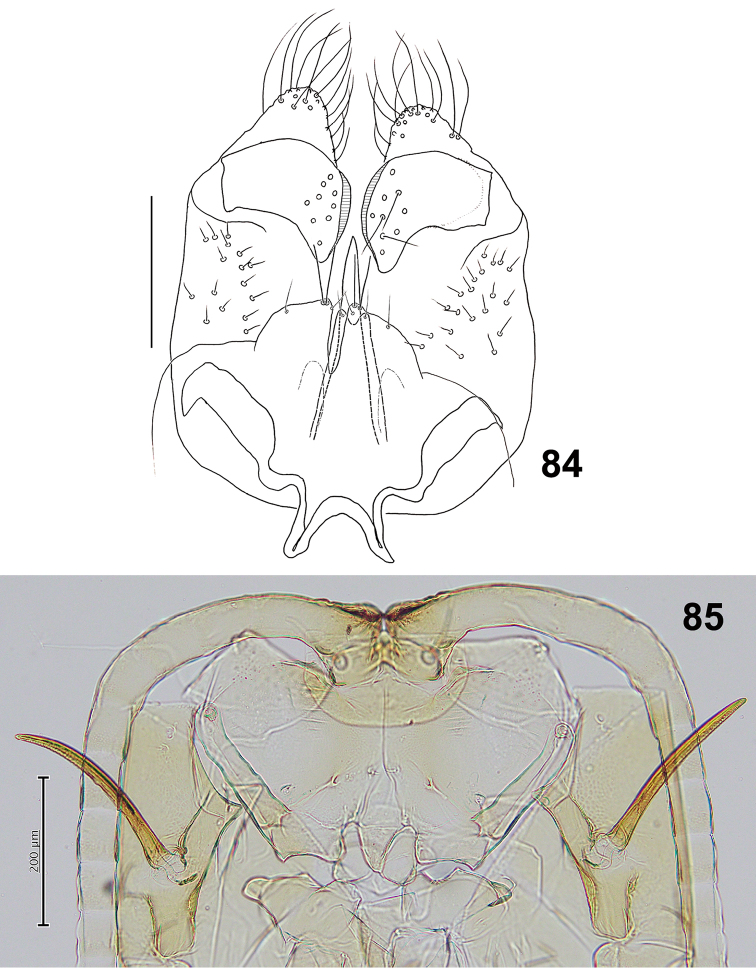
*Schizomyiapaederiae*. **84** Male terminalia **85** Ventral view of pupal head and prothorax. Scale bars: 50 µm (**84**), 200 µm (**85**).

*Male abdomen*: Trichoid sensilla present on sternites II–VIII in median position, except on VIII in lateral position. Terminalia (Fig. 84): Gonocoxite length about 3.3 times as long as gonostylus. Gonostylus with slim tooth in the dorsal and ventral views.

*Mature larva*: Abdominal segment VIII without dorsal papillae. Two groups of lateral papillae on all thoracic segments, each consisting of 2 setose and 1 asetose papillae. The terminal segment with 1 setose and 6 asetose terminal papillae (Yukawa 1983).

*Pupa* (Fig. 85): Prothoracic spiracle 260–290 μm long (*n* = 5).

###### Distribution.

Japan: Hokkaido, Honshu and Shikoku (Yukawa and Masuda 1996).

###### Remarks.

This species had been described by Shinji (1938) under the genus *Schizomyia*. Then, Yukawa (1983) combined the species with *Asteralobia* because of its shallowly constricted male flagellomeres. Because *Asteralobia* is synonymized under *Schizomyia* in this paper, *S.patriniae* is combined again with *Schizomyia*.

*Schizomyiapatriniae* is distinguishable from known *Schizomyia* species, except three species that were previously treated as *Asteralobia* and newly combined here under *Schizomyia*, i.e. *S.sasakii*, *S.soyogo*, and *S.humuli*, by the presence of shallow constrictions on male flagellomeres and the absence of corniform papillae on the terminal larval segment. *S.patriniae* can be easily separated from *S.sasakii*, *S.soyogo* and *S.humuli* based on the number of papillae on the larval terminal segment: *S.patriniae* possesses two setose and six asetose terminal papillae, but *S.sasakii* and *S.soyogo* have only six setose terminal papillae, and *S.humuli* has four setose terminal papillae.

##### Schizomyia asteris

Taxon classificationAnimaliaDipteraCecidomyiidae

(Kovalev, 1964) comb. n.

Figs 86–93; Table S4

Asteralobia asteris Kovalev, 1964

Characters as in *S.achyranthesae* except for the following:

###### Material examined.

2♂, 2♀: (Mamaev collection: slide no. B1-251363 & 251364), galls collected from *Aster* sp. in Kedrovaja Pad reserve, Russian Far East on 28.viii.1964. 4 larvae: galls collected from *A.tataricus* in Smolyaninovo, Primorsky Territory, Russian Far East on 13.ix.2002, M. Tokuda leg. 6 larvae: galls collected from *A.scaber* in Smolyaninovo, Primorsky Territory, Russian Far East on 13.ix.2002, M. Tokuda leg.

###### Description.

*Head*: Compound eyes with rounded facets; facets on the vertex and eye bridge unobservable. Palpus: first segment ca 34.5 μm, second 1.8 times as long as the first, third 1.2 as long as the second, fourth 1.1 as long as the third.

**Figures 86–93.**
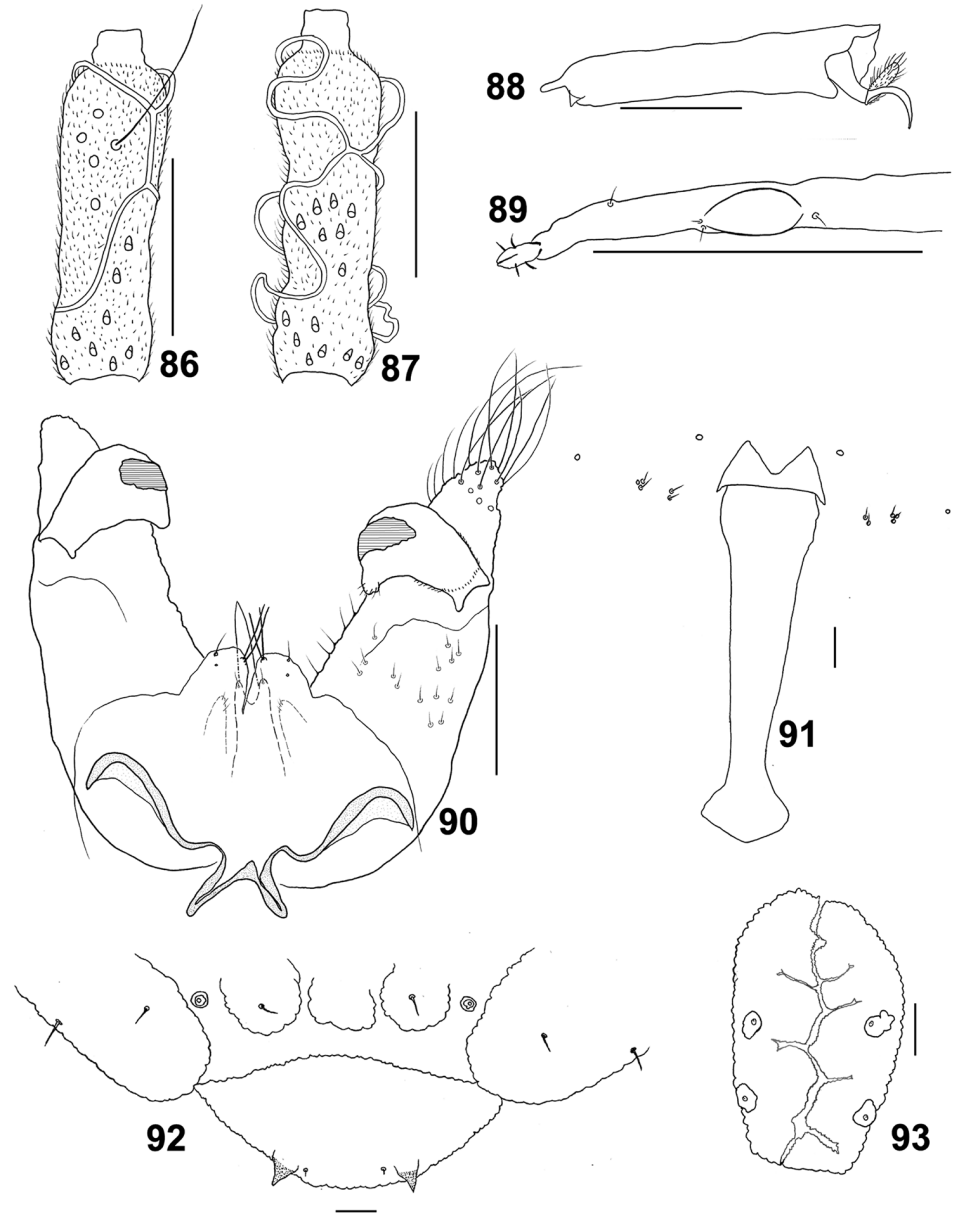
*Schizomyiaasteris*. **86** Lateral view of female flagellomere V **87** Lateral view of male flagellomere V **88** Tarsomere V and acromere **89** Ovipositor apex **90** Male terminalia **91** Larval spatula **92** Terminal larval segments dorsally **93** Larval anus. Scale bar: 50 µm.

*Thorax*: Wing length 1.93 mm (*n* = 1) in female, 1.55–2.02 mm (*n* = 2) in male. Anepimeral setae 14–15 (*n* = 3); mesanepisternum scales 17–23 (*n* = 7); lateral scutum setae 23–29 (*n* = 4). Lengths of leg segments as in Suppl. material 1: Table S4.

*Female abdomen*: Ovipositor: protrusible needle-like portion about 5.7 times as long as sternite VII; cerci divided medially, with sclerotized margins and few setae (Fig. 89).

*Male abdomen*: Terminalia (Fig. 90): Gonocoxite with pointed apical lobe extending beyond gonostylus. Gonocoxite length about 3.5 times as long as gonostylus. Gonostylus without setae ventrally and dorsally.

*Mature larva*: Sternal spatula with posterior portion about 3.3 times as wide as the base of the anterior free portion (Fig. 91). Abdominal segment VIII with three dorsal lobes, the outer two each with 1 setose dorsal papilla (Fig. 92). Anus with branched opening, and 4 asetose anal papillae (Fig. 93).

*Pupa*: Prothoracic spiracle about 220 μm long (*n* = 1).

###### Distribution.

Russian Far East (Kovalev 1964; Tokuda et al. 2003).

###### Remarks.

Although Kovalev (1964) mentioned that *S.asteris* is closest to *S.doellingeriae*, Tokuda et al. (2003) showed that they are distinguishable by the number of lateral papillae, terminal papillae, and the shape of anal opening.

**Figures 94–95.**
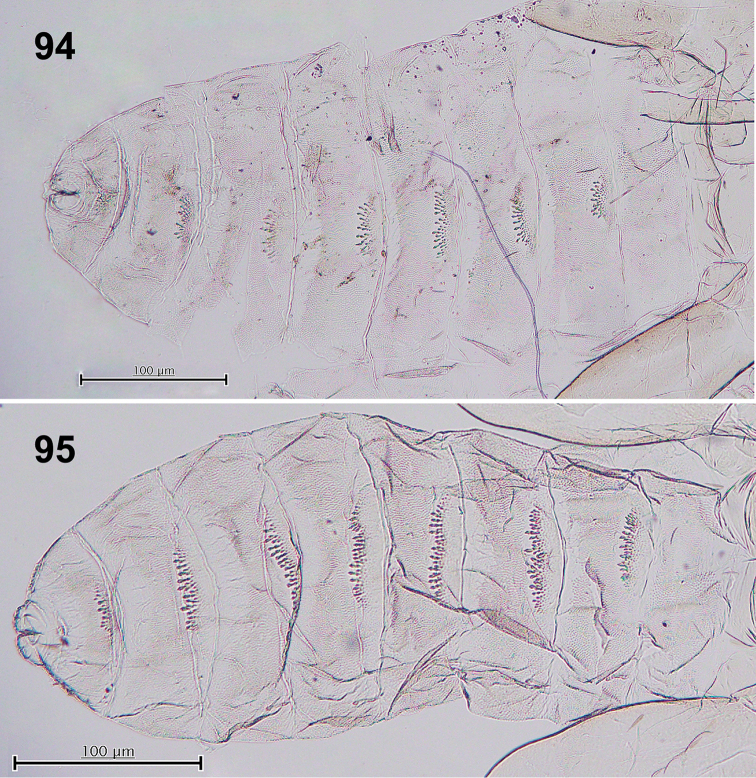
Dorsal view of pupal abdominal segments. **94***Schizomyiasoyogo***95***Schizomyiasasaki*. Scale bar: 100 µm.

**Figure 96.**
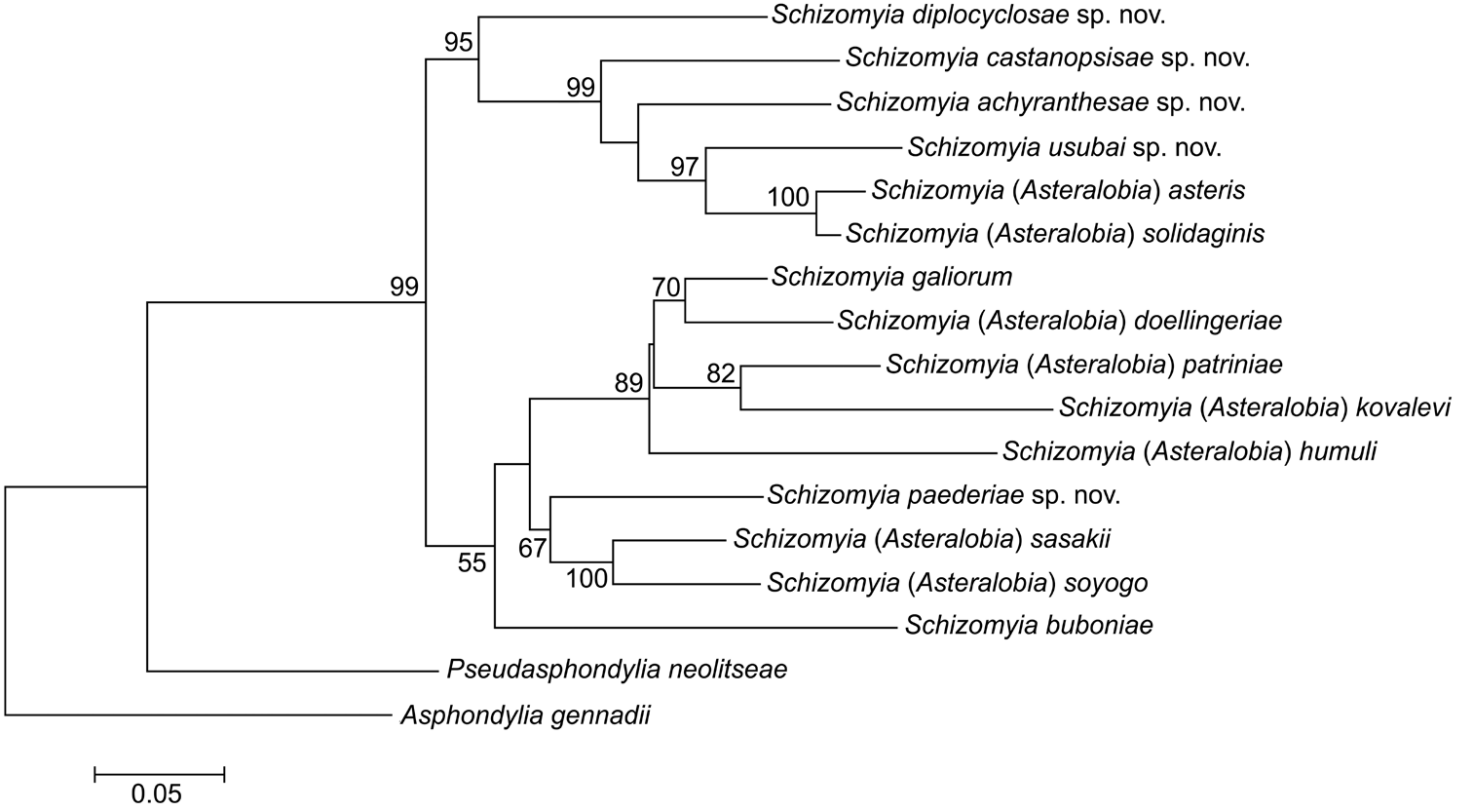
A phylogenetic reconstruction based on partial sequences of cytochrome oxidase subunit I (COI) and 12S small ribosomal subunit genes. The topology and branch length were produced by the maximum likelihood method (note the scale bar). Bootstrap values are indicated at branches gaining more than 50% support (10^3^ replications).

##### Taxonomic key to *Schizomyia* species in Japan

**Table:** 

1	Male flagellomeres deeply constricted (Fig. 58)	***S.paederiae* sp. n.**
–	Male flagellomeres shallowly constricted (Fig. 7)	**2**
2	Trichoid sensilla present medially on adult sternites II–VI	**3**
–	Trichoid sensilla present laterally anterior to the sclerotized sternites II–VI in adults	**7**
3	Larval terminal segment with 8 terminal papillae	**4**
–	Larval terminal segment with fewer than 8 terminal papillae	**5**
4	Terminal papillae of 2 corniform and 6 setose papillae (Tokuda et al. 2003: fig. 1A)	*** S. doellingeriae ***
–	Terminal papillae of 2 setose and 6 asetose (Yukawa 1983: fig. 4E)	*** S. paterinia ***
5	Terminal papillae made up of 4 setose papillae: 2 with long setae and 2 with tiny setae	*** S. humuli ***
–	Terminal papillae made up of 6 setose papillae	**6**
6	Pupal dorsal abdominal spines covering about 1/4 of the upper area of terga II–VIII (Fig. 94) (see Tokuda et al. 2004 for full-description of the species)	*** S. soyogo ***
–	Pupal dorsal abdominal spines covering about 1/3 of the upper area of terga II–VIII (Fig. 95) (see Tokuda et al. 2004 for full-description of the species)	*** S. sasakii ***
7	Larval anal opening simple (e.g. Fig. 14)	**8**
–	Larval anal opening branched (e.g. Fig. 40)	**9**
8	Protrusible needle-like portion of ovipositor about 4 times as long as sternite VII (Fig. 10)	***S.achyranthesae* sp. n.**
–	Protrusible needle-like portion of ovipositor about 3 times as long as sternite VII (Fig. 23)	***S.diplocyclosae* sp. n.**
9	Protrusible needle-like portion of ovipositor about 3.3 times as long as sternite VII (Fig. 35)	***S.castanopsisae* sp. n.**
–	Protrusible needle-like portion of ovipositor about 4.5 times as long as sternite VII (Fig. 49)	***S.usubai* sp. n.**

## Molecular phylogenetic study

The complete molecular dataset of COI and 12S consisted of approximately 800 bp. The monophyly of *Schizomyia* was strongly supported with a 99% bootstrap value, and the genus was divided into two main clades. One clade with a 55% bootstrap support was subdivided into three subclades: one including *S.galiorum*, *S.doellingeriae*, *S.humuli*, *S.patriniae* and *S.kovelavi*; another including *S.sasakii, S.soyogo*, and *S.paederia*; and the third comprising *S.buboniae*. The second main clade contains six morphologically-close species: *S.diplocyclosae*, *S.castanopsisae*, *S.achyranthesae*, *S.solidaginis*, *S.asteris* and *S.usubai*, and gained a 95% bootstrap support.

## Discussion

In the present study, we showed that constricted male flagellomeres, the only character used to separate *Asteralobia* from *Schizomyia* (Kovalev 1964), can be also observed in the type species of *Schizomyia*, *S.galiorum*, and hence *Asteralobia* is synonymized here under *Schizomyia*. Our molecular phylogenetic analysis strongly supported this conclusion with high bootstrap values.

Because of the broad definition of *Schizomyia*, which depends only on plesiotypic characters (Gagné and Marohasy 1997; Gagné 1994), some other genera of Schizomyiina, e.g., *Metasphondylia*, *Placochela* and *Schizandrobia*, are considered to fit easily into its definition (Gagné and Jaschhof 2017). Comprehensive taxonomic and molecular analyses including these genera are needed for further progress on the taxonomy of *Schizomyia*.

In the present study, six eastern Palearctic *Schizomyia* species, namely *S.achyranthesae, S.asteris*, *S.diplocyclosae*, *S.castanopsisae*, *S.usubai* and *S.solidaginis*, were shown to be close to each other and (although we have never examined the phylogenetic position of *S.solidaginis*) constructed a monophyletic clade in the molecular analysis. They differ from all known *Schizomyia* spp. by the laterally situated anterior pair of trichoid sensilla, which are present anterior to the sclerotized sternite. This character can be considered as derived because in other genera of Schizomyiina, the anterior pair of trichoid sensilla are usually located on the sternites. Future comprehensive taxonomic studies may treat these species as a natural cluster within *Schizomyia*.

Several important characters need to be re-evaluated in order to meet current taxonomic standards in many *Schizomyia* species. For example, the ovipositors of most known *Schizomyia* species were not described in detail, although they can be expected to be variable because of their adaptation for oviposition on different organs of hosts belonging to various, not related, families. Similarly, the pupa, which offers many diagnostic features for taxonomy in Schizomyiina (Möhn 1961), is still unknown and undescribed in many species, especially in those that develop in the soil and are not easily found. Descriptions of these unknown morphological features of *Schizomyia* species are essential to clarify the generic concept.

## Supplementary Material

XML Treatment for Schizomyia achyranthesae

XML Treatment for Schizomyia diplocyclosae

XML Treatment for Schizomyia castanopsisae

XML Treatment for Schizomyia usubai

XML Treatment for Schizomyia paederiae

XML Treatment for Schizomyia galiorum

XML Treatment for Schizomyia patriniae

XML Treatment for Schizomyia asteris

## Supplementary materials

Supplementary material 1 Supplementary data Data type: measurements Explanation note: **Table S1.** Length (μm) of each leg segment in *Schizomyiaachyranthesae* and *S.diplocyclosae*. **Table S2.** Length (μm) of each leg segment in *Schizomyiatokudai* and *S.usubai*. **Table S3.** Length (μm) of each leg segment in *Schizomyiapaederiae* and *S.galiorum*. **Table S4.** Length (μm) of each leg segment in *Schizomyiapatriniae* and *S.asteris*. This is an open access article distributed under the terms of the Creative Commons Attribution License (CC BY 4.0), which permits unrestricted use, distribution, and reproduction in any medium, provided the original author and source are credited.Ayman Khamis Elsayed, Junichi Yukawa, Makoto Tokuda
