# Supplementary material for: A taxonomic revision and molecular phylogeny of the eastern Palearctic species of the genera Schizomyia Kieffer and Asteralobia Kovalev (Diptera, Cecidomyiidae, Asphondyliini), with descriptions of five new species of Schizomyia from Japan
Source: Zookeys. 2018 Dec 18;(808):123–60. doi: 10.3897/zookeys.808.29679 (PMC6305770; doi:10.3897/zookeys.808.29679)
Supplement: Supplementary material 1 — Supplementary data [file zookeys-808-123-s001.docx]

Table S1. Length (μm) of each leg segment in *Schizomyia achyranthesae* and *S. diplocyclosae*.

| **Species** |  | ***Schizomyia achyranthesae*** | | | | ***Schizomyia diplocyclosae*** | | | |
| --- | --- | --- | --- | --- | --- | --- | --- | --- | --- |
| **Sex** |  | **Female (n=4)** | | **Male (n=3)** | | **Female (n=3)** | | **Male (n=3)** | |
| **Leg** |  | **Mean** | **Range** | **Mean** | **Range** | **Mean** | **Range** | **Mean** | **Range** |
| **Fore leg** | **Femur** | 700 | 620–750 | 680 | 650-700 | 773.3 | 760–780 | 784.3 | 760-810 |
|  | **Tibia** | 725 | 680–760 | 730 | 710-750 | 780 | 760–800 | 751.7 | 665-810 |
|  | **Tarsomere I** | 97.5 | 90–100 | 97.7 | 95–100 | 110 | 110 | 103 | 100–107 |
|  | **Tarsomere II** | 567.5 | 490-600 | 558.3 | 540–590 | 613.3 | 610–620 | 590 | 580–600 |
|  | **Tarsomere III** | 380 | 310-440 | 346.3 | 335–364 | 333.3 | 320–350 | 295 | 280–310 |
|  | **Tarsomere IV** | 247.5 | 200–280 | 201.3 | 194–210 | 216.7 | 210–230 | 200 | 175–225 |
|  | **Tarsomere V** | 151.3 | 135–160 | 133.3 | 120–150 | 156.7 | 150–165 | 140 | 120–160 |
| **Mid leg** | **Femur** | 670 | 620–720 | 630 | 620–660 | 733.3 | 710–760 | 687.3 | 670–710 |
|  | **Tibia** | 637.5 | 590–670 | 623.3 | 610–640 | 699 | 680–735 | 665 | 630–690 |
|  | **Tarsomere I** | 96.3 | 95–100 | 94 | 90–100 | 100 | 110 | 101 | 100–103 |
|  | **Tarsomere II** | 468.8 | 405–500 | 470 | 450–500 | 476.7 | 460–500 | 485 | 480–490 |
|  | **Tarsomere III** | 328.8 | 280–350 | 241.7 | 295–330 | 476.7 | 260–290 | 193.3 | 280–300 |
|  | **Tarsomere IV** | 198.75 | 165–230 | 185 | 180–195 | 191.7 | 185–200 | 185 | 180–190 |
|  | **Tarsomere V** | 130 | 110–150 | 117 | 110–121 | 140 | 130–150 | 120 | 105–135 |
| **Hind leg** | **Femur** | 810 | 750–850 | 716.7 | 650–770 | 926.7 | 870–1000 | 853.3 | 750–950 |
|  | **Tibia** | 747.5 | 690–800 | 693.3 | 630–730 | 830.3 | 781–860 | 803.3 | 750–840 |
|  | **Tarsomere I** | 100 | 90–105 | 93.3 | 90–95 | 110.3 | 106–115 | 95.7 | 85–102 |
|  | **Tarsomere II** | 592.5 | 500–650 | 601.7 | 560–625 | 641.7 | 570–685 | 641 | 670–610 |
|  | **Tarsomere III** | 393.8 | 325–450 | 348.3 | 315–380 | 348.3 | 320–375 | 350 | 340–360 |
|  | **Tarsomere IV** | 247.5 | 200–290 | 226.7 | 220–240 | 230.7 | 210–240 | 287.5 | 220–355 |
|  | **Tarsomere V** | 140 | 130–160 | 125 | 110–140 | 161.3 | 150–170 | 152.5 | 130–175 |

Table S2. Length (μm) of each leg segment in *Schizomyia tokudai* and *S. usubai*.

| **Species** |  | ***Schizomyia tokudai*** | | | | ***Schizomyia usubai*** | | | |
| --- | --- | --- | --- | --- | --- | --- | --- | --- | --- |
| **Sex** |  | **Female (n=3)** | | **Male (n=3)** | | **Female (n=3)** | | **Male (n=3)** | |
| **Leg** |  | **Mean** | **Range** | **Mean** | **Range** | **Mean** | **Range** | **Mean** | **Range** |
| **Fore leg** | **Femur** | 1035 | 950–1130 | 1018.3 | 970–1055 | 829.3 | 720–888 | 706.7 | 610-760 |
|  | **Tibia** | 998.3 | 875–1070 | 1067 | 1021–1110 | 846 | 735–903 | 758.3 | 625-840 |
|  | **Tarsomere I** | 115 | 105–130 | 113.3 | 105–125 | 102 | 93–103 | 93.7 | 80–101 |
|  | **Tarsomere II** | 736.7 | 675–785 | 770 | 730–800 | 680 | 680 | 583.3 | 500–650 |
|  | **Tarsomere III** | 426.7 | 365–465 | 435 | 410–465 | 400 | 400 | 321.7 | 250–360 |
|  | **Tarsomere IV** | 290 | 250–310 | 278.7 | 246–300 | 360 | 360 | 216.7 | 160–250 |
|  | **Tarsomere V** | 180 | 165–190 | 178 | 164–190 | 190 | 190 | 136.7 | 110–130 |
| **Mid leg** | **Femur** | 931.7 | 915–960 | 845 | 760–960 | 764 | 672–820 | 665 | 600–725 |
|  | **Tibia** | 825 | 690–930 | 880 | 820–940 | 716.7 | 620–780 | 746.7 | 570–1010 |
|  | **Tarsomere I** | 122.3 | 117–130 | 111.5 | 103–120 | 105.7 | 97–110 | 92.7 | 80–100 |
|  | **Tarsomere II** | 543 | 495–590 | 595 | 590–600 | 477.3 | 400–520 | 423 | 390–650 |
|  | **Tarsomere III** | 322.3 | 285–360 | 355.5 | 351–360 | 273.3 | 230–310 | 293.3 | 200–400 |
|  | **Tarsomere IV** | 205 | 185–230 | 281.5 | 223–240 | 186.7 | 165–200 | 220 | 120–350 |
|  | **Tarsomere V** | 158.3 | 150–165 | 156 | 154–160 | 137.7 | 118–155 | 130 | 100–160 |
| **Hind leg** | **Femur** | 1055 | 900–1215 | 1064 | 1032–1100 | 921.3 | 840–984 | 840 | 720–950 |
|  | **Tibia** | 975 | 825–1055 | 1028.7 | 981–1070 | 853.3 | 720–920 | 661.7 | 525–800 |
|  | **Tarsomere I** | 122 | 155–130 | 119.3 | 113–125 | 111.7 | 95–130 | 91.7 | 80–100 |
|  | **Tarsomere II** | 715 | 660–770 | 858 | 824–880 | 650 | 540–750 | 546.7 | 510–620 |
|  | **Tarsomere III** | 441.5 | 418–465 | 480.7 | 472–520 | 382 | 324–420 | 320 | 300–360 |
|  | **Tarsomere IV** | 269 | 250–288 | 310 | 300–330 | 244 | 212–260 | 200 | 180–240 |
|  | **Tarsomere V** | 250 | 155–190 | 180 | 165–200 | 170.7 | 150–182 | 123.3 | 110–140 |

Table S3. Length (μm) of each leg segment in *Schizomyia paederiae* and *S. galiorum*.

| **Species** |  | ***Schizomyia paederiae*** | | | | ***Schizomyia galiorum*** | | | |
| --- | --- | --- | --- | --- | --- | --- | --- | --- | --- |
| **Sex** |  | **Female (n=3)** | | **Male (n=2)** | | **Female (n=4)** | | **Male (n=2)** | |
| **Leg** |  | **Mean** | **Range** | **Mean** | **Range** | **Mean** | **Range** | **Mean** | **Range** |
| **Fore leg** | **Femur** | 516.7 | 390–600 | 511 | 470-552 | 515 | 462–583 | 554 | 468–640 |
|  | **Tibia** | 534.3 | 400–601 | 515 | 470-560 | 487 | 316–591 | 530 | 580–479 |
|  | **Tarsomere I** | 80 | 70–90 | 72 | 70–74 | 68 | 56–75 | 90 | 87–93 |
|  | **Tarsomere II** | 505 | 450–560 | 380 | 380 | 282 | 254-316 | 374 | 374 |
|  | **Tarsomere III** | 195 | 160–230 | 185 | 185 | 164 | 133–191 | 184 | 184 |
|  | **Tarsomere IV** | 150 | 140–160 | 120 | 120 | 109 | 95–123 | 149 | 149 |
|  | **Tarsomere V** | 100 | 90–110 | 80 | 80 | 66 | 61–70 | 85 | 85 |
| **Mid leg** | **Femur** | 463.7 | 350–541 | 465 | 420-510 | 482 | 395–673 | 552 | 420–683 |
|  | **Tibia** | 430 | 330–490 | 460 | 430–490 | 443 | 354–533 | 525 | 433–617 |
|  | **Tarsomere I** | 80 | 70–90 | 72.5 | 70–75 | 83 | 73–90 | 81 | 77–84 |
|  | **Tarsomere II** | 290 | 250–330 | 330 | 300–360 | 267 | 184–348 | 375 | 301–412 |
|  | **Tarsomere III** | 157.5 | 130–185 | 174 | 150–198 | 169 | 137–212 | 187 | 132–241 |
|  | **Tarsomere IV** | 105 | 90–120 | 117.5 | 110–125 | 102 | 79–151 | 118 | 91–145 |
|  | **Tarsomere V** | 97.5 | 90–105 | 77 | 74–80 | 82 | 63–90 | 71 | 55–87 |
| **Hind leg** | **Femur** | 603.3 | 490–670 | 561.5 | 510–613 | 631 | 518–743 | 531 | 518-543 |
|  | **Tibia** | 531.7 | 380–615 | 540 | 500–580 | 536 | 410–672 | 508 | 497–518 |
|  | **Tarsomere I** | 86 | 70–98 | 75 | 70–82 | 91 | 73–109 | 83 | 78–87 |
|  | **Tarsomere II** | 405 | 360–450 | 460 | 460 | 262 | 241–279 | 316 | 308–323 |
|  | **Tarsomere III** | 230 | 185–275 | 280 | 280 | 186 | 151–222 | 174 | 141–206 |
|  | **Tarsomere IV** | 160 | 115–205 | 196 | 196 | 137 | 111–203 | 105 | 99–111 |
|  | **Tarsomere V** | 97.5 | 80–115 | 110 | 110 | 112 | 77–150 | 96 | 80–111 |

Table S4. Length (μm) of each leg segment in *Schizomyia patriniae* and *S. asteris*.

| **Species** |  | ***Schizomyia patriniae*** | | | | ***Schizomyia asteris*** | | | |
| --- | --- | --- | --- | --- | --- | --- | --- | --- | --- |
| **Sex** |  | **Female (n=4)** | | **Male (n=2)** | | **Female (n=2)** | | **Male (n=2)** | |
| **Leg** |  | **Mean** | **Range** | **Mean** | **Range** | **Mean** | **Range** | **Mean** | **Range** |
| **Fore leg** | **Femur** | 750 | 710–780 | 665 | 640–690 | 715 | 692–737 | 820 | 814–825 |
|  | **Tibia** | 788.8 | 750–825 | 630 | 560–700 | 557 | 386–727 | 748 | 409 |
|  | **Tarsomere I** | 108 | 100–117 | 95.5 | 94–97 | 105 | 104–105 | 147 | 114–180 |
|  | **Tarsomere II** | 458.8 | 535–625 | 467.5 | 445–490 | 542 | 542 | 589 | 598 |
|  | **Tarsomere III** | 327.5 | 300–340 | 344 | 335–353 | 332 | 332 | 297 | 297 |
|  | **Tarsomere IV** | 218 | 200–230 | 160 | 150–170 | 239 | 239 | 281 | 281 |
|  | **Tarsomere V** | 141.3 | 135–145 | 117 | 109–125 | 126 | 126 | 97 | 97 |
| **Mid leg** | **Femur** | 735 | 710–810 | 600 | 570–630 | 677 | 666–687 | 732 | 732 |
|  | **Tibia** | 738.8 | 650–900 | 550 | 500–600 | 497 | 407-586 | 616 | 616 |
|  | **Tarsomere I** | 107 | 93–113 | 97.5 | 95–100 | 118 | 111–125 | 100 | 100 |
|  | **Tarsomere II** | 483 | 400-640 | 356 | 340–370 | 318 | 232–404 | -- | -- |
|  | **Tarsomere III** | 286.3 | 240–370 | 105.5 | 190–210 | 248 | 325–261 | -- | -- |
|  | **Tarsomere IV** | 195 | 160–250 | 135 | 130–140 | 187 | 187 | -- | -- |
|  | **Tarsomere V** | 128.8 | 120–150 | 100 | 90–110 | 132 | 132 | -- | -- |
| **Hind leg** | **Femur** | 862.5 | 800–900 | 730 | 710–750 | 717 | 578–855 | 784 | 632–936 |
|  | **Tibia** | 823.8 | 800–850 | 682.5 | 650–715 | 644 | 522–765 | 759 | 606–912 |
|  | **Tarsomere I** | 112.5 | 105–120 | 93.5 | 92–95 | 87 | 64–110 | 110 | 86–133 |
|  | **Tarsomere II** | 587.5 | 530–630 | 497.5 | 485–510 | 408 | 332–483 | 561 | 561 |
|  | **Tarsomere III** | 337.5 | 310-360 | 280 | 270–290 | 229 | 167–290 | 478 | 478 |
|  | **Tarsomere IV** | 230 | 210–250 | 182.5 | 175–190 | 159 | 146–171 | 226 | 226 |
|  | **Tarsomere V** | 142 | 130–153 | 122 | 110–120 | -- | -- | 161 | 161 |
